# Effects of a three-month exercise programme on cognition, mood and neurogenesis: the NeuroFit randomised controlled trial

**DOI:** 10.1038/s41514-026-00439-w

**Published:** 2026-09-17

**Authors:** Sahand Farmand, Curie Kim, Andrea Du Preez, André O. Werneck, Lucia Batzu, Dominic Pearce, Kirsten Brady, Markos Khir, Sarah Nicolas, John F. Cryan, Yvonne M. Nolan, Brendon Stubbs, Sandrine Thuret

**Affiliations:** 1 Department of Basic and Clinical Neuroscience, Institute of Psychiatry, Psychology and Neuroscience, King’s College London, London, UK; 2 Department of Psychological Medicine, Institute of Psychiatry, Psychology and Neuroscience, King’s College London, London, UK; 3 Department of Anatomy and Neuroscience, University College Cork, Cork, Ireland; 4 APC Microbiome Ireland, University College Cork, Cork, Ireland; 5 Clinical Division of Social Psychiatry, Department of Psychiatry and Psychotherapy, Medical University of Vienna, Vienna, Austria; 6 Comprehensive Center for Clinical Neurosciences and Mental Health (C3NMH), Medical University of Vienna, Vienna, Austria; 7 Department of Psychiatry and Neurosciences, Charité Campus Mitte, Charité, Universitätsmedizin Berlin, Berlin, Germany; 8 Division of Psychology and Mental Health, Manchester Academic Health Science Centre, University of Manchester, Manchester, UK

**Keywords:** Neurology, Neuroscience

## Abstract

Middle age represents a critical window for mental and cognitive change, yet it remains relatively understudied as a target period for preventative lifestyle interventions. We conducted a three-month randomised controlled trial in healthy middle-aged adults in the UK (*n* = 52) to examine the effects of exercise on pattern separation, cognition and well-being, blood-based biomarkers, and hippocampal neurogenesis, using an in vitro parabiosis assay. No significant intervention-by-time effect was observed for the primary outcome, pattern separation (*β* = 0.33, *p* = 0.26). However, significant intervention-by-time effects were detected for depressive symptoms, as measured by the PHQ-9 (*β* = −1.18, adjusted *p* = 0.011), and for the percentage of proliferative cells in vitro (*β* = −0.23, adjusted *p* = 0.02), suggesting that exercise remodels the circulating milieu in ways that directly influence hippocampal progenitor biology. Overall, our findings suggest that a structured, instructor-led, live online exercise programme in midlife is feasible and may produce temporally staged adaptations, with early effects on mood, intermediate systemic biological changes influencing hippocampal progenitor dynamics, and cognitive benefits that may require longer exposure to emerge. The trial was prospectively registered at Clinicaltrials.gov (NCT05397990).

## Introduction

Middle age, typically defined as the period between 40 and 65 years, is increasingly recognised as a vulnerable phase for mental health and emotional well-being. Large population-based studies demonstrate that subjective well-being and mood often decline during midlife, with a higher prevalence of depressive symptoms and psychological distress compared with earlier and later adulthood^1^. Notably, these changes coincide with major social and occupational transitions that can lead to reduced levels of physical activity^2^, which may in turn adversely affect quality of life and overall health. Midlife also marks a critical window for both structural and functional brain changes^3–5^. Yet, despite its significance, this life stage remains understudied, both as a potential predictor of cognitive decline and as a target period for preventive lifestyle interventions.

Among brain regions, the hippocampus has long been recognised for its central role in cognition, particularly in spatial and episodic memory^6^. It is also sensitive to the ageing process, exhibiting non-linear changes across the lifespan, with pronounced alterations often beginning or becoming more apparent during the fifth and sixth decades of life^7,8^. As a result, the hippocampus has become a focal point of studies aimed at understanding and potentially mitigating age-related cognitive decline^9^. In humans, cost-effective lifestyle interventions, particularly physical exercise, have demonstrated the potential to attenuate age-associated hippocampal atrophy and increase hippocampal volume in healthy individuals and those with neurodegenerative diseases^10,11^. In addition to its structural effects, exercise has been associated with enhancements in hippocampal-dependent cognition, including spatial memory^10^, and pattern separation^12^, a process by which the brain discriminates between similar, overlapping stimuli and new information. Pattern separation is understood to be dependent on adult hippocampal neurogenesis (AHN)^13^, a process through which new functional neurons arise from the neural stem cells located within the dentate gyrus of the hippocampus^14–18^. Interestingly, in vivo studies have demonstrated that AHN is sensitive to exercise^19^, supporting the hypothesis that exercise-induced modulation of hippocampal cognition occurs through regulation of AHN. Although the exact mechanisms remain under investigation, it is hypothesised that physical exercise enhances AHN and hippocampal-dependent cognition through circulating blood-borne factors^20^, alongside remodelling of the neurogenic vasculature and increased secretion of growth and antioxidant meidators^21^.

**Fig. 1:**
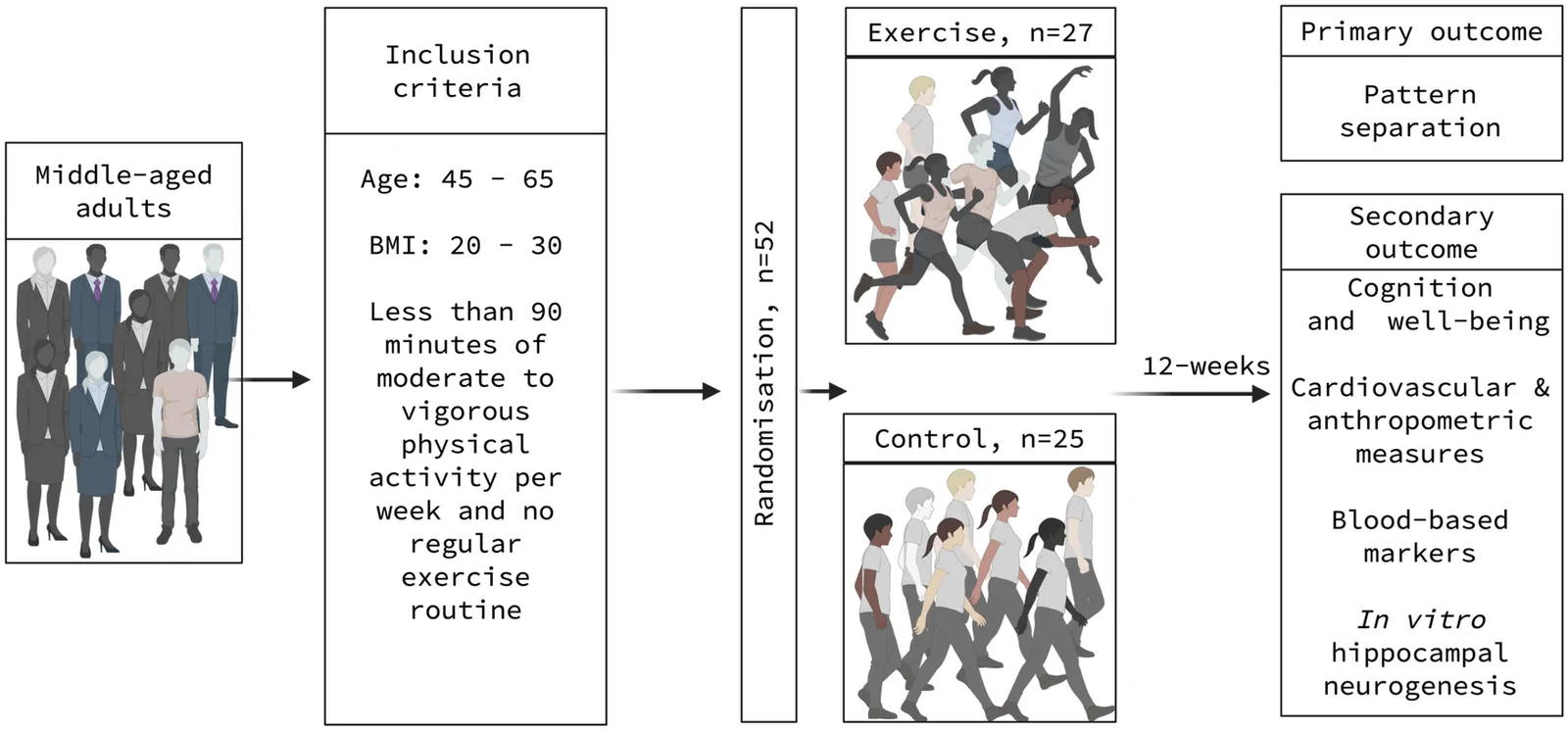
NeuroFit randomised controlled trial. The figure outlines the key stages of the NeuroFit intervention trial. Middle-aged participants aged 45–65 years, with a BMI between 20 and 30 and engaging in less than 90 min of physical activity per week, were recruited (additional exclusion criteria are detailed in the Participants section) and randomised to either an exercise group (*n* = 27) or a control group (*n* = 25). Participants in the exercise group completed a 12-week structured exercise programme, while those in the control group continued their usual daily activities. Outcomes were assessed at baseline and at the endpoint (week 12). The primary outcome was pattern separation. Secondary outcomes included measures of cognition and well-being, cardiovascular and anthropometric indices, blood-based biomarkers, and hippocampal neurogenesis, assessed using an in vitro parabiosis assay (Figure created using bioRender).

At present, no non-invasive method exists to directly measure AHN in humans. Consequently, exercise-induced structural changes within the dentate gyrus^22^ and functional outcomes such as pattern separation^12^ are commonly used as proxy measures of exercise-induced AHN. To date, only a small number of studies have examined the effects of exercise on pattern separation in humans^12,23–26^. However, these studies have typically employed short-term intervention designs and have focused primarily on younger or older adults. To our knowledge, no previous randomised controlled trial (RCT) has examined the effects of a chronic exercise intervention on hippocampal-dependent cognition in midlife while simultaneously integrating a translational human model of AHN. Given evidence for ongoing brain alterations during midlife, the NeuroFit RCT was designed to examine whether a three-month physical exercise intervention could improve pattern separation in middle-aged adults. In addition to this primary objective, we aimed to determine whether the intervention influenced broader indicators of mental and physical well-being, including cognitive and mood measures, cardiovascular and anthropometric indices, blood-based biomarkers, and hippocampal neurogenesis, using an in vitro parabiosis assay (Fig. 1).

## Results

**Fig. 2:**
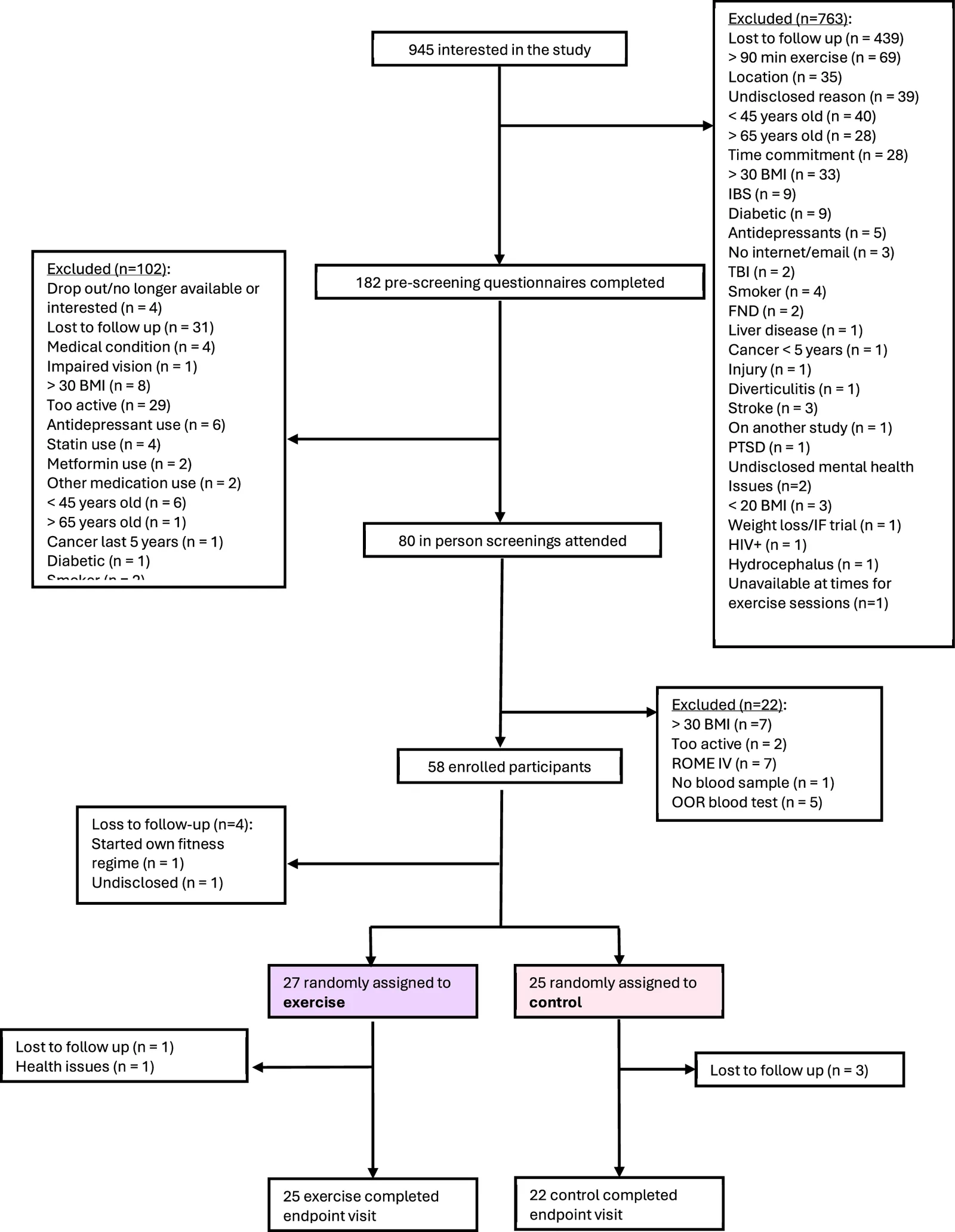
Consort diagram (Intention-to-treat trial profile). The flow diagram illustrates participant progression through the trial, including screening, enrolment, randomisation, and endpoint assessment.

Between March 29, 2022, to June 12, 2024, a total of 945 individuals expressed interest in taking part in the NeuroFit trial. Of these, 763 were excluded due to not meeting the inclusion criteria, residing outside the study catchment area, or being lost to follow up. Among the 182 individuals who completed the pre-screening questionnaire, 80 attended the in-person screening visits. Of those, 58 met the eligibility criteria and were enrolled in the study. Following additional dropouts prior to trial commencement, the final enrolled sample consisted of 52 individuals (Fig. 2). Following randomisation, 27 individuals were assigned to the exercise group and 25 to the control group. Of these, 25 (93%) and 22 (85%) individuals, respectively, attended the endpoint visit (Fig. 2). Additionally, participants in the exercise group attended a mean of 86% (SD 13.79; range 42–100%) of all prescribed exercise sessions. Analysis of primary and secondary outcomes were conducted on an intention-to-treat basis, including all randomised participants (*n* = 27 for the exercise; *n* = 25 for the control group; Fig. 2) and per-protocol analysis, including those who had no major deviations from the trial (*n* = 35). Mean age of the 52 participants in the study was 55.1 years (SD 6.1), of whom 37 (71%) were female and 15 (29%) males (Table 1). Self-reported demographics in our study suggest a more diverse population compared with the UK average, with White British being underrepresented and overrepresentation of other ethnicity groups^27^. No serious adverse events were observed during the trial, and no withdrawals were considered related to the exercise intervention.

**Table 1:** Baseline Characteristics of the intention-to-treat population

CHARACTERISTIC	CONTROL *N* = 25^a^	EXERCISE *N* = 27^a^	OVERALL *N* = 52^a^
Age (years)	56.7 (5.2)	53.7 (6.5)	55.1 (6.1)
Sex			
Female	17 (68%)	20 (74%)	37 (71%)
Male	8 (32%)	7 (26%)	15 (29%)
Ethnicity			
Asian or Asian British—Chinese	0 (0%)	1 (3.7%)	1 (1.9%)
Asian or Asian British—Indian	1 (4.0%)	1 (3.7%)	2 (3.8%)
Black or Black British—African	2 (8.0%)	2 (7.4%)	4 (7.7%)
Black or Black British—Caribbean	1 (4.0%)	1 (3.7%)	2 (3.8%)
Mixed—multiple ethnic groups	1 (4.0%)	0 (0%)	1 (1.9%)
Other ethnic groups—Other	1 (4.0%)	3 (11%)	4 (7.7%)
White—British/Welsh/Scottish/Northern Irish	13 (52%)	13 (48%)	26 (50%)
White—Irish	0 (0%)	2 (7.4%)	2 (3.8%)
White—Other	6 (24%)	4 (15%)	10 (19%)
Education			
Secondary	3 (12%)	3 (11%)	6 (12%)
Bachelor’s degree	7 (28%)	9 (33%)	16 (31%)
Master’s degree	8 (32%)	7 (26%)	15 (29%)
Doctorate degree	2 (8.0%)	2 (7.4%)	4 (7.7%)
Other	5 (20%)	6 (22%)	11 (21%)

^a^Mean (SD); *n* (%).

### Primary outcome

**Fig. 3:**
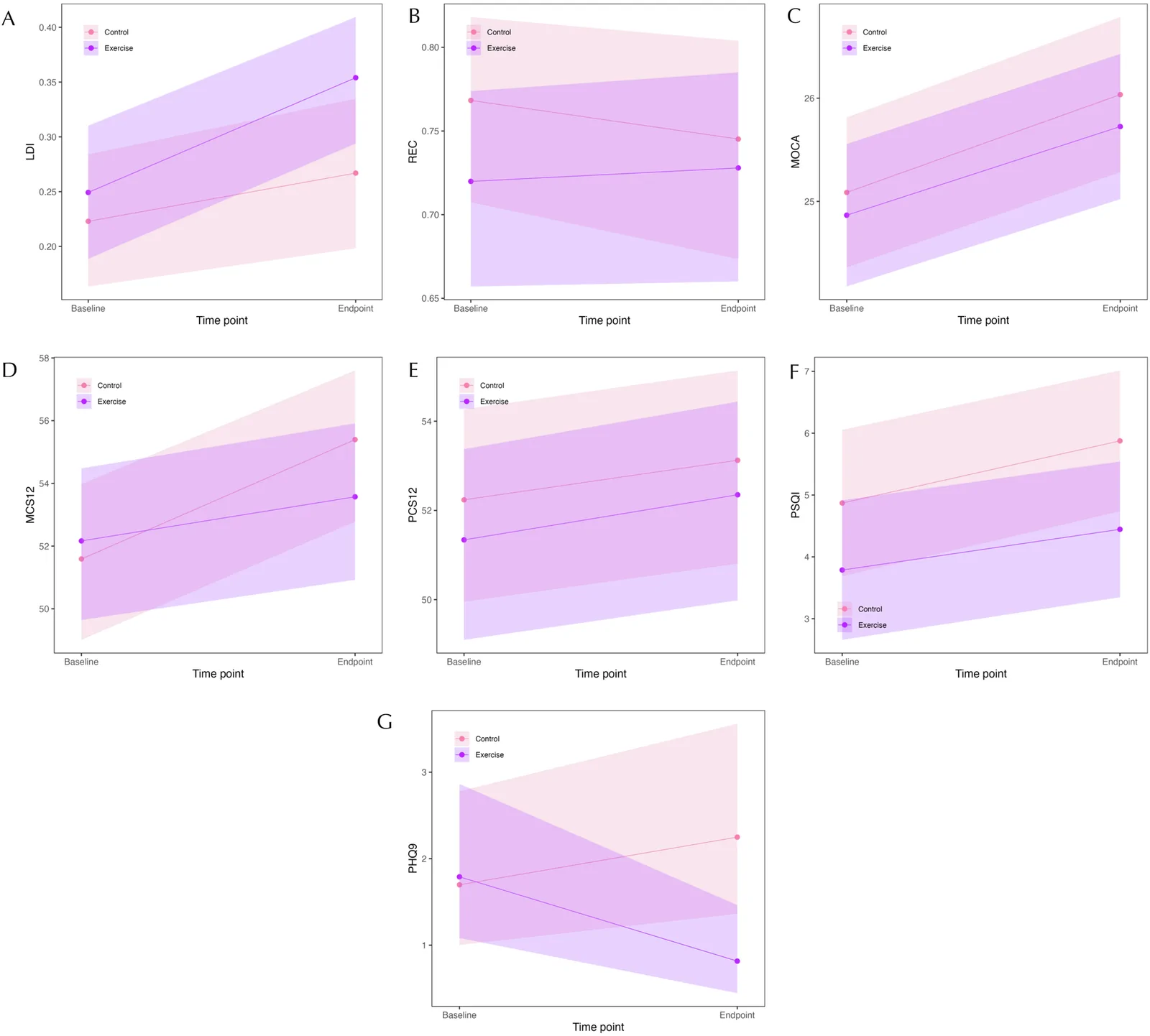
Estimated marginal means of the primary and mental and physical well-being outcomes at baseline and endpoint by intervention group. Points represent adjusted mean values for the exercise and control groups at each time point, with shaded areas indicating 95% confidence intervals. Estimates are adjusted for age, sex, years of education, and baseline outcome, and account for repeated measurements within participants. The figure illustrates changes in outcomes over time within each group and the between-group differences at baseline and endpoint. **A** Lure discrimination index, LDI. **B** Recognition memory, REC. **C** Montreal cognitive assessment, MoCA. **D** Mental component of short form questionnaire, MCS12. **E** Physical component of short form questionnaire, PCS12. **F** Pittsburgh sleep quality index, PSQI. **G** Patient health questioannire-9, PHQ-9.

Intention-to-treat analysis revealed no significant interaction of intervention by time on the primary outcome, LDI (*n* = 52, *β* = 0.33, SE = 0.29, 95% CI −0.24 to 0.89, *p* = 0.26, Fig. 3A). Similarly, we observe no significant effect of intervention by time on our primary outcome in the per-protocol population (*n* = 35, *β* = 0.27, SE = 0.37, 95% CI −0.46 to 1.01, *p* = 0.47; Supplementary Table 1).

### Secondary cognitive and well-being outcomes

Intention-to-treat analysis showed a significant interaction of intervention by time on IPAQ category (*n* = 52, *β* = 4.18, SE = 1.21, 95% CI 1.82 to 6.55, adjusted *p* = 0.0007). We observed no significant interaction of intervention by time on recognition memory (*n* = 52, *β* = 0.18, SE = 0.29, 95% CI −0.39 to 0.76, adjusted *p* = 0.61; Fig. 3B), MoCA (*n* = 52, *β* = −0.089, SE = 0.60, 95% CI −1.30 to 1.12, adjusted *p* = 0.96; Fig. 3C), MCS12 (*n* = 52, *β* = −0.33, SE = 0.28, 95% CI −0.89 to 0.22, adjusted *p* = 0.39; Fig. 3D), PCS12 (*n* = 52, *β* = 0.011, SE = 0.25, 95% CI −0.49 to 0.51, adjusted *p* = 0.97; Fig. 3E) or PSQI (*n* = 52, *β* = −0.35, SE = 0.80, 95% CI −1.96 to 1.26, adjusted *p* = 0.76; Fig. 3F). Further per-protocol analysis was in line with the intention-to-treat analysis, demonstrating no significant effect of intervention by time on the named outcomes. Among the secondary outcomes, we observed a significant effect of intervention by time on the PHQ-9 score in the intention-to-treat analysis population (*n* = 52, *β* = −1.18, SE = 0.40, 95% CI −1.95 to −0.41, adjusted *p* = 0.011; Fig. 3G), however this effect was weakened in the per-protocol-population (*n* = 35, *β* = −1.21, SE = 0.48, 95% CI −2.14 to −0.27, adjusted *p* = 0.047; Supplementary Table 1).

### Secondary clinical and blood-based outcomes

Although a positive trend was observed for percentage body fat, this effect did not withstand correction for multiple comparisons. Overall, no significant intervention-by-time interactions were detected for any of the secondary clinical or blood-based outcomes (Table 2).

**Table 2:** Indicates the results (estimates (95% CI), standard error, *p* value and the adjusted *p* value), for intervention by time for select secondary clinical and blood-based outcomes, for the intention-to-treat population

Intention-to-treat		Intervention*time			
Outcomes	*n*	Estimate (95% CI)	Standard error	*p*	Adjusted *p*
Secondary clinical outcomes					
Resting HR (BPM)	52	−0.26 (−4.99; 4.46)	2.35	0.91	0.96
HR (BPM) post 3’ step test	52	3.92 (−0.72; 8.56)	2.31	0.097	0.20
Weight	52	−0.18 (−1.25; 0.89)	0.53	0.74	0.86
Waist to hip ratio	52	0.0075 (−0.025; 0.040)	0.016	0.65	0.96
BMI	52	−0.078 (−0.46; 0.30)	0.19	0.68	0.98
Body fat (%)	52	0.071 (0.0019; 0.14)	0.035	0.044	0.071
Systolic BP	52	−2.50 (−8.12; 3.11)	2.78	0.37	0.50
Diastolic BP	52	1.33 (−2.73; 5.40)	2.02	0.51	0.59
Secondary blood-based outcomes					
Serum BDNF (ng/mL)	52	1.18 (−1.35; 3.72)	1.26	0.35	0.77
Serum Klotho (ng/mL)	50	−0.070 (−0.45; 0.31)	0.19	0.71	0.96
Plasma Clusterin (μg/mL)	50	6.99 (−5.01; 19.01)	5.94	0.25	0.53

The interaction term (*) represents the difference in change over time between the exercise and control groups and has been adjusted for age, sex, years of education and baseline differences. The *p*-value for the secondary outcomes has been adjusted using the Benjamini–Hochberg false discovery rate method.

*HR* Heart rate, *BMI* Body mass index, *BP* Blood pressure, *BDNF* brain derived neurotrophic factor.

### Secondary neurogenesis outcomes during proliferation

**Fig. 4:**
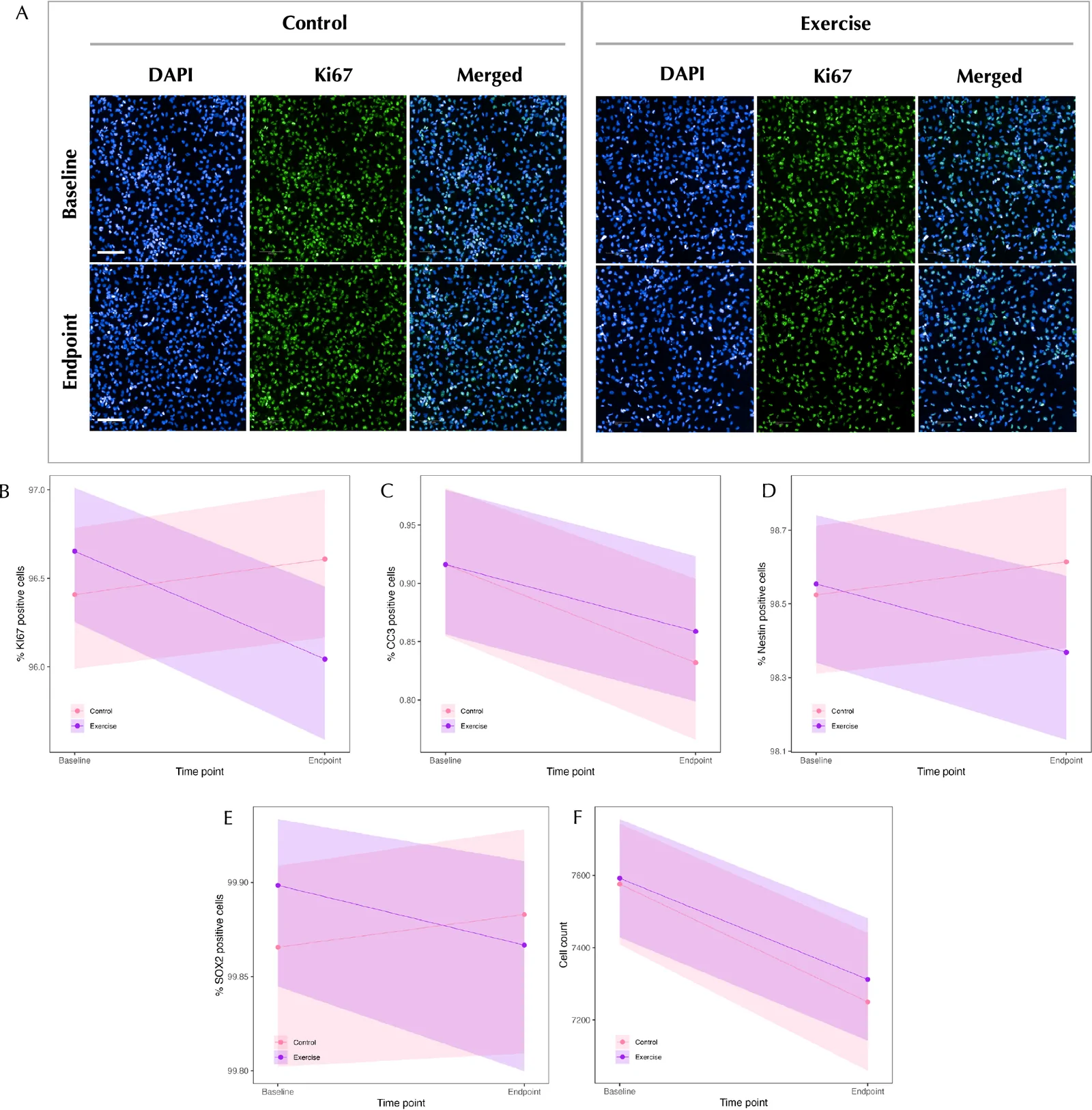
Effect of serum from participants in the exercise and control groups on expression of neurogenesis associated markers in HPC0A07/03 A cells, during the proliferation phase, in vitro. **A** Representative immunostaining images show cells treated with participant serum collected at baseline and at endpoint. (20 x water objective; Blue: DAPI; nuclear marker, Green: Ki67; cell proliferation marker). All experiments were performed in three biological replicates, each comprising three technical replicates. Scale bar, on the bottom left represents 100 mM. **B** Estimated marginal means of Ki67 at baseline and endpoint by intervention group. Points represent adjusted mean values for the exercise and control groups at each time point, with shaded areas indicating 95% confidence intervals. Estimates are adjusted for baseline outcome and batch differences. **C** Estimated marginal means of CC3 at baseline and endpoint by intervention group are adjusted for baseline outcome and batch differences. **D** Estimated marginal means of Nestin at baseline and endpoint by intervention group are adjusted for baseline outcome and batch differences. **E** Estimated marginal means of Sox2 at baseline and endpoint by intervention group are adjusted for baseline outcome and batch differences. **F** Estimated marginal means of cell count at baseline and endpoint by intervention group are adjusted for baseline outcome and batch differences. Abbreviations; Ki67, Kiel-67 antigen; cell proliferation marker; CC3, Cleaved Caspase-3, apoptotic marker; Nestin, neuroepithelial stem cell protein, progenitor integrity marker; Sox2, SRY-box transcription factor 2, stem cell ness marker.

To assess secondary neurogenesis outcomes in the intention-to-treat population, we focussed on markers relevant to the neurogenic process. We observed a small but statistically significant intervention-by-time interaction in the percentage of cells expressing Ki67, a marker of cell proliferation (*n* = 52, *β* = −0.23, SE = 0.09, 95% CI −0.41 to −0.06, adjusted *p* = 0.02; Fig. 4.A, B). No significant intervention-by-time effects were detected for other proliferation stage markers, including the percentage of CC3 positive cells, a marker of apoptosis-mediated cell death (*n* = 52, *β* = 0.03, SE = 0.07, 95% CI −0.10 to 0.16, adjusted *p* = 0.93; Fig. 4C), percentage of Nestin positive cells, a marker of progenitor integrity (*n* = 52, *β* = −0.19, SE = 0.12, 95% CI −0.42 to 0.05, adjusted *p* = 0.18; Fig. 4D), the percentage of Sox2 positive cells, also indicative of progenitor integrity (*n* = 52, *β* = -0.41, SE = 0.34, 95% CI −1.07 to 0.25, adjusted *p* = 0.31; Fig. 4E), or total cell number (*n* = 52, *β* = 46, SE = 150.43, 95% CI −257.33 to 349.43, adjusted *p* = 0.76; Fig. 4F). Results from the per-protocol analysis were consistent, showing a similar significant reduction in Ki67 positive cells with no differences in expression of other neurogenesis-associated markers (Supplementary Table 2).

### Secondary neurogenesis outcomes during differentiation

We observed no significant interaction of intervention by time for any of the secondary neurogenesis outcomes, during the differentiation stage (Table 3).

**Table 3:** Indicates the results (estimates (95% CI), standard error, *p* value and the adjusted *p* value), for intervention by time for secondary neurogenesis outcomes during proliferation and differentiation phase, for the intention-to-treat analysis

Intention-to-treat		Intervention*time			
Secondary neurogenesis outcomes	*n*	Estimate (95% CI)	Standard error	*p*	Adjusted *p*
Differentiation stage					
%Ki67 positive cells	52	0.0079 (−0.043; 0.059)	0.026	0.76	0.85
%CC3 positive cells	52	0.0031 (−0.12; 0.12)	0.062	0.96	0.96
%DCX positive cells	52	−0.17 (−0.33; −0.0080)	0.083	0.040	0.089
%MAP2 positive cells	52	−0.055 (−0.22; 0.11)	0.083	0.50	0.57
Neurite length (px)	52	−0.012 (−0.26; 0.24)	0.12	0.92	0.92
Cell number	52	−109 (−360; 144)	126	0.39	0.70

The interaction term (*) represents the difference in change over time between the exercise and control groups and has been adjusted for baseline differences and immunostaining batch. The *p*-value for the secondary neurogenesis outcomes has been adjusted using the Benjamini–Hochberg false discovery rate method.

*Ki67* Kiel-67 antigen, cell proliferation marker, *CC3* Cleaved Caspase-3, apoptotic marker, Nestin; neuroepithelial stem cell protein; progenitor integrity marker, *Sox2* SRY-box transcription factor 2; stem cell ness marker, *DCX* Doublecortin; immature neurons marker, *MAP2* Microtubule-Associated Protein 2; mature neurons marker.

## Discussion

To the best of our knowledge, the NeuroFit RCT is the first trial in middle-aged adults to evaluate the effects of a sustained physical exercise programme on pattern separation, cognition and broader mental and physical well-being, while simultaneously integrating a translational in vitro model that investigates exercise-induced AHN. Contrary to our hypothesis, intention-to-treat and per-protocol analyses of our primary outcome, revealed no statistically significant improvement in pattern separation (as measured by LDI) in the exercise group compared with controls upon completion of the 3-month trial. As expected, our analysis demonstrated a significant increase in physical activity levels for the participants in the exercise group over time, reflected by a greater progression to higher IPAQ categories. Among the secondary outcomes, the intention-to-treat analysis showed a significant reduction in depressive symptoms, as measured by the PHQ-9, in the exercise group compared with controls over time. Meanwhile, we observed no other significant intervention related changes in any other of the cognitive, well-being, clinical and blood-based secondary outcomes. When assessing secondary neurogenesis outcomes, we observed that serum collected from participants in the exercise group significantly reduced the percentage of proliferative cells in vitro at endpoint versus baseline, whereas levels remained unchanged in controls, suggesting that exercise-altered circulating factors could influence cellular behaviour in vitro.

Our findings for the primary outcome stand in contrast to the view that exercise regulates hippocampal-dependent cognition, particularly, pattern separation^12,24,26,28^. To date, very few RCTs with a between-subject design have examined this question. A recent RCT in healthy adolescents and young adults also reported no exercise-induced improvement in pattern separation, underscoring the challenge of demonstrating durable effects in this domain^23^. Most positive evidence instead derives from within-subject crossover studies, in which each participant serves as their own control, thereby reducing variability and increasing sensitivity to short-term effects, but limiting conclusions about longer-term change. Second, positive findings have largely been reported in young adults^12^ or older individuals^29^, where single bouts of light-to-moderate exercise transiently improved mnemonic discrimination^24^. These findings raise the idea that responsiveness to exercise, in the context of pattern separation, may depend on age, with younger adults benefiting from heightened neural plasticity and older adults showing greater short-term gains due to increased hippocampal vulnerability. By contrast, cognitively and physically healthy individuals with minimal comorbidity, such as those enrolled in our trial, likely begin near the upper bound of hippocampal-dependent task performance, leaving limited room for measurable improvement. In such samples, both biological and psychometric ceiling effects may attenuate observable change, such that longer duration and/or higher intensity interventions may be required to induce durable alterations in hippocampal function.

The absence of improvement in pattern separation after three months of exercise in our trial may also reflect the time required for hippocampal adaptations to accumulate. Prior studies have demonstrated that exercise increases hippocampal volume and memory performance after 6–12 months of training^10^, whereas shorter interventions generally show limited effects in healthy adults^30^. Consistent with this, we observed no change in circulating BDNF, a neurotrophic factor often upregulated by exercise and central to hippocampal plasticity^21,31^. The lack of a BDNF response provides a plausible mechanistic explanation for the absence of improvement in hippocampal-dependent cognition, suggesting that longer duration or higher intensity training may be needed to elicit both neurotrophic and cognitive benefits.

Finally, a further consideration is the timing of outcome assessment. In our trial, the endpoint LDI was measured within a week of the final exercise session. By contrast, acute crossover studies reporting positive effects have consistently administered the MST immediately after a single bout of exercise when transient neurobiological changes such as increased catecholamines, arousal, and attention are most pronounced^32^. These state-dependent effects likely sharpen dentate gyrus computations under conditions of high interference, yielding short-lived improvements in pattern separation. Such gains, however, may not accumulate into durable adaptations detectable at later time points, particularly in cognitively healthy, middle-aged adults.

The observed significant improvement in mood, reflected in reduced depressive symptoms in the exercise group, compared with controls, is consistent with previous studies^33,34^. Notably, participants in our study were neither clinically nor sub-clinically depressed at baseline, with PHQ-9 scores falling within the minimal symptom range. Despite this, exercise led to a greater reduction in PHQ-9 scores than controls. Although, a 5-point decrease on the PHQ-9 is commonly considered as clinically significant in treatment settings^35,36^, such criteria were developed primarily for individuals with established depressive symptoms and may not be directly applicable to non-clinical populations. In samples where baseline scores are already low, the dynamic range for improvement is inherently constrained, limiting the magnitude of observable change. Consequently, modest changes, such as those observed here, suggest that exercise can promote psychological well-being even among healthy, middle-aged adults, a life stage recognised as vulnerable to mood disturbances^1^. This is important as even small improvements in mood in non-depressed populations are relevant for healthy longevity, as subclinical symptoms are associated with diminished quality of life and increased risk of later cognitive decline^37^. Importantly, converging evidence suggests that, in non-clinical populations, the benefits of exercise may be better conceptualised as preventative rather than therapeutic, shifting depressive symptoms away from clinically relevant thresholds over time. For example, De Zeeuw and colleagues demonstrated in a workplace-based RCT that a brief supervised exercise programme reduced the proportion of employees exceeding subthreshold depression cut-offs, effectively lowering risk status despite modest changes in mean symptom scores^38^. Similarly, another study^34^ reported that mixed-mode exercise can reduce the occurrence of PHQ-9 defined depressive episodes during follow-up despite participants not being recruited for major depression, supporting the role of exercise in preventing emergent symptoms rather than treating established disorder. Within this context, even small reductions in depressive symptoms, as observed here, may represent meaningful population-level benefits, contributing to the maintenance of psychological health in midlife.

Our findings from the in vitro parabiosis neurogenesis assay are in line with previous in vivo work, showing that circulating factors are biologically active and can alter the neurogenic process^39^. Our findings indicate that exposure to serum derived from exercisers, relative to serum from control participants, did not alter progenitor integrity, neuronal differentiation, or total cell number. However, it was associated with a selective reduction in the proportion of proliferating cells, as measured by percentage of Ki67 positive cells during the proliferation phase. The absence of change in the proportion of apoptotic cells, a feature that typically accompanies reduced Ki67 expression in inflammatory or cytotoxic in vitro context^40^, suggests that the modest decrease in proliferation observed here does not reflect a deleterious or anti-proliferative milieu. Similarly, the stability of Nestin and Sox2 positive populations, established markers of neural stem and progenitor cell identity, indicates preservation of the progenitor pool and argues against depletion or compromised stem cell integrity.

Although these findings offer valuable insight into how exercise-induced circulating factors influence hippocampal progenitor dynamics, they should be interpreted cautiously. The in vitro neurogenesis assay employed here, although experimentally controlled and mechanistically informative, represents a reductionist system and cannot fully capture the complexity of the in vivo neurogenic niche, where cellular behaviour is shaped by dynamic interactions among systemic physiology, vascular signalling, local microenvironmental cues, and neural activity. Consequently, any biological interpretation must be considered within the constraints of this model.

Within these limitations, several biologically plausible mechanisms may nonetheless account for the selective reduction in Ki67 positive cells observed following exposure to exerciser-derived serum. First, the effect may reflect a form of homeostatic cell-cycle restraint associated with long-term training adaptations. Rodent studies consistently demonstrate that the proliferative response to exercise and voluntary running is temporally dynamic, with early increases in progenitor proliferation that attenuate or normalise with continued exercise exposure^41–43^. These findings suggest that sustained physical exercise may progressively shift the neurogenic response away from rapid progenitor expansion and towards mechanisms supporting survival, maturation, and functional integration of newly generated neurons. Under such conditions, circulating factors present at rest may no longer carry a strong pro-mitotic signal and could even favour reduced cell-cycle entry, consistent with the modest decrease in proliferative fraction observed in our assay.

Second, the composition and modality of the exercise intervention may have influenced the systemic milieu. Experimental evidence in rodents indicates that sustained aerobic exercise is the most potent driver of AHN, whereas resistance training alone exerts comparatively weaker or more variable effects on progenitor proliferation^44^. Our mixed-modality programme, which distributed time across aerobic and resistance components, may therefore have produced systemic adaptations that promote general metabolic and psychological health without specifically enhancing mitogenic signalling to neural progenitors. Such a profile could plausibly explain the absence of increased proliferation in vitro.

Third, it is important to recognise that circulating serum primarily reflects chronic physiological adaptation rather than the transient biochemical fluctuations that occur immediately following physical exercise. Acute bouts of physical exercise are known to induce short-lived increases in circulating neurotrophic and growth-related factors in an intensity-dependent manner^21,45^. However, these exerkine responses are typically transient and return to baseline within hours^46–48^. In contrast, serum collected under resting conditions, particularly when temporally removed from the most recent exercise bout, is generally considered to reflect longer-term systemic adaptations rather than transient post-exercise signalling. Consistent with this, serum samples in the present study were obtained within one week of the final exercise session and were therefore temporally distant from the immediate post-exercise window. As such, these samples likely capture chronic physiological states, including broader homeostatic regulations. Consequently, the in vitro milieu generated by exerciser-derived serum may more closely represent a stable, regulated physiological environment than an acutely pro-neurogenic or pro-proliferative stimulus. This interpretation is also consistent with the absence of detectable differences in circulating BDNF between exercisers and controls following completion of the trial.

Finally, the findings may be cautiously interpreted through a quiescence preservation framework^49^, an increasingly recognised principle in the regulation of adult neural stem cell pools. In vivo, excessive or sustained activation of progenitors can accelerate stem cell depletion, whereas quiescence-maintaining programmes act to safeguard long-term neurogenic capacity and tissue homeostasis^21^. Consistent with this, rodent studies indicate that antiproliferative regulators can constrain stem cell activation even in the context of PE, particularly with advancing age^50^, suggesting that heightened neurogenic activity is not indefinitely sustained but instead tightly regulated over time. Although our model employs a fetal-derived progenitor line rather than the adult subgranular zone niche, a serum-driven reduction in the proportion of cycling cells may nonetheless reflect systemic signals that favour maintenance and stability over continued expansion of the progenitor pool. Within this framework, reduced Ki67 expression would not necessarily indicate impaired neurogenic potential, but rather a subtle recalibration of cell cycle dynamics toward preservation of stem cell integrity and longer-term resilience. Together, these findings suggest that the reduced proliferative fraction reflects adaptive regulation rather than detrimental suppression of neurogenesis.

An interpretation of the present findings is that the effects of exercise on the hippocampus and behaviour follow a temporally staged trajectory rather than emerging simultaneously across domains. Improvements in mood may represent an early and sensitive signal of exercise responsiveness, reflecting rapid psychosocial changes. Intermediate adaptations may occur at the level of systemic biology, where repeated training progressively reshapes circulating molecular profiles capable of influencing cellular behaviour, particularly hippocampal progenitors, as suggested by the serum-mediated effects observed in our neurogenesis assay. By contrast, measurable improvements in hippocampal-dependent cognition may represent a later-emerging outcome, requiring more prolonged or intensive exposure before structural and network-level neural adaptations accumulate sufficiently to influence behavioural performance. Such a phased model, in which affective, systemic, and cognitive responses unfold on partially distinct timescales, may help reconcile the presence of mood and biological changes in the absence of detectable cognitive improvement after three months of training, and suggests that intervention duration and cumulative exercise dose are critical determinants.

Our study has several notable strengths. Adherence to the intervention was high, with a mean attendance rate of 86%. This is particularly important given that participants were recruited based on low baseline physical activity, and it suggests that our exercise programme was well tolerated. This level of engagement also underscores the value of co-designed interventions that align with participant motivations and preferences, ensuring that exercise programmes are both acceptable and sustainable in real-world settings. Our strict inclusion and exclusion criteria, and especially the restriction to individuals with low self-reported physical activity, enhance internal validity by ensuring that observed intervention-related effects can be more confidently attributed to the exercise programme itself. The marked increase in physical activity levels in the exercise group further supports the effectiveness of the design and the targeted recruitment approach. Additionally, we integrated a comprehensive battery of cognitive, psychological, physiological, and biomarker outcomes, allowing us to assess exercise effects across multiple domains of healthy ageing. Finally, we incorporated a translational in vitro model of AHN, providing unique mechanistic insight into the systemic effects of exercise and enabling us to bridge behavioural and cellular levels of analysis. Together, these features strengthen the interpretability of our findings and highlight the translational potential of a pragmatic exercise intervention in healthy and understudied middle-aged population.

However, our study also had some limitations that should be acknowledged. Our intervention was designed for three months, which may be insufficient to induce durable changes in hippocampal-dependent cognition, particularly given evidence that such adaptations often emerge after 6–12 months of training. Notably, the cohort was predominantly female (71%), and although self-reported ethnicity suggested a more diverse population than the UK average, subgroup sizes were too small to explore potential sex or ethnicity-related differences in intervention response. Participants were also generally healthy at baseline (Supplementary Tables 3 and 4), which may limit the generalisability of our findings to populations with greater comorbidity or clinical burden. At the same time, this relatively healthy profile may have reduced our ability to detect meaningful changes in well-being outcomes, given the potential for ceiling effects. Thus, caution is warranted when generalising our findings to the wider population. Importantly, although no serious adverse events were reported, the absence of adverse outcomes in this relatively healthy, sample cannot be extrapolated to individuals with comorbidities or higher baseline activity. Additionally, while our in vitro neurogenesis assay provides important mechanistic insight, it cannot fully recapitulate the complexity of AHN in vivo, and our findings should be interpreted as indicative rather than definitive evidence of systemic effects on the AHN. Furthermore, exercise-induced circulating factors have been shown to modulate inflammatory signalling within the hippocampus^51^. The absence of inflammatory marker measurements in the present study therefore limits our ability to determine whether inflammation-related pathways contributed to the observed AHN-related outcomes. Future studies incorporating inflammatory biomarkers alongside functional measures of AHN will be important for clarifying the interplay between exercise, inflammation, and neurogenesis.

Finally, future research should aim to address the heterogeneity of response to exercise. In our trial, we observed variability across participants in the exercise group, with some showing improvements in cognition (LDI; Supplementary Fig. 1) and/or mood (PHQ-9; Supplementary Fig. 2), others showing little or no change, and a minority showing declines. Importantly, a comparable pattern was also evident in our in vitro assay across multiple cellular markers, particularly in the proportion of Ki67 positive cells (Supplementary Fig. 3). HPCs exposed to serum from exercisers showed divergent changes in this proliferation marker, with some samples exhibiting reductions (consistent with the intervention*group interaction), while others showed minimal change or increases. The convergence of behavioural and cellular variability suggests that responsiveness to exercise may reflect underlying biological differences rather than random variation alone. Contemporary molecular exercise frameworks demonstrate that systemic responses to exercise are multi-layered, and exhibit marked inter-individual variability^52^ and within a precision prevention framework^53^, such heterogeneity represents an opportunity. Integrating circulating molecular profiles with cellular readouts may enable identification of response endotypes and predictive biomarkers of neurocognitive benefit, shifting the field from estimating average treatment effects toward biologically informed targeting of lifestyle interventions for brain health.

In summary, the NeuroFit trial shows that a pragmatic, tolerable exercise programme is feasible for healthy, physically inactive middle-aged adults and can meaningfully improve mood and increase physical activity levels. The online group-based format achieved high attendance, indicating promise for scalable community and home-based models. The absence of detectable effects on pattern separation suggests that hippocampal plasticity may require a higher cumulative dose of exercise than is needed for early mood benefits, particularly in individuals with normal baseline cognitive functioning. Our study also provides one of the first human demonstrations that physical exercise can remodel the circulating milieu in a manner that influences hippocampal progenitor biology in vitro. These findings position NeuroFit as an important first step in understanding how exercise may influence neurocognitive ageing in midlife, and they underscore the need for longer or more intensive interventions, and potentially multimodal approaches, to determine whether exercise can produce durable neurocognitive benefits. Together with our in vitro observations, the results highlight that systemic effects of exercise on hippocampal plasticity may require extended exposure to emerge, reinforcing the value of midlife as a critical window for preventive strategies.

## Methods

### Study design

NeuroFit was an open label, two-arm, parallel group, randomised controlled trial including healthy middle-aged adults in the UK. Participants were randomly assigned to one of two groups: “Exercise” or “Control”. Participants completed two study visits: one at baseline (within 1 week of commencing the exercise program) and one at end point (within 1 week of finishing the 12-week program); an optional follow up visit was conducted at week 24. Ethics approval was obtained from the Health Faculties Research Ethics Subcommittee at King’s College London (Project Reference: HR/DP-21/22-28287). The trial was registered at Clinicaltrials.gov (NCT05397990) on 20/05/2022 and is completed. The study was conducted in accordance with the Declaration of Helsinki. All subjects provided written informed consent before any procedures were carried out.

### Participants

Participants were recruited through both internal and external advertisements. Internal recruitment involved the use of posters and email circulars distributed within King’s College London and King’s Health Partners. For external recruitment, posters were placed in community establishments likely to attract the target demographic. Additionally, advertisements were published in relevant local newspapers and via social media. Participants interested in the study could register via telephone or email. Upon registration, they were provided with study information and were asked to complete eligibility questionnaires. Individuals meeting the initial eligibility criteria were invited to attend a screening visit, where written informed consent was obtained. At this visit, participants reported their ethnicity and sex, with male and female response options. Blood pressure and anthropometric measurements (weight, height, waist circumference, body fat percentage and BMI) were recorded, and participants were instructed to complete the International Physical Activity Questionnaire (IPAQ)^54^, Mini-Mental State Examination (MMSE)^55^, ROME IV^56^. Participants also completed the Mnemonic Similarity Task (MST)^57^, to ensure all have the same level of familiarity with the test prior to baseline visit. Finally, a fasted blood sample was collected (Supplementary Note 1; details of blood handling) to assess full blood count, plasma glucose, liver function, and lipid profile. Those who were eligible based on results from the screening visit, were formally invited to attend a baseline visit.

Inclusion criteria were as follows: (1) adults aged 45–65 years, to focus on changes occurring in midlife; (2) a body mass index (BMI) between 20 and 30; and (3) engagement in less than 90 min of moderate to vigorous physical activity per week, with no regular exercise routine, as determined by self-report.

Participants were excluded if they were unable to provide informed consent or complete cognitive testing, had uncorrected visual impairment, were pregnant or lactating, smoked, or were unwilling or unable to engage in structured exercise (including failure to meet physical activity readiness questionnaire criteria or unwillingness to exercise three times weekly). Additional exclusions included current or recent (past 2 years) psychiatric disorders; neurological or neurodevelopmental conditions affecting cognition; significant cardiovascular, respiratory, gastrointestinal, metabolic, or liver disease; cancer within the past 5 years; abnormal screening blood results; alcohol misuse; sleep disorders or shift work; and use of medications or supplements known to affect cognition, mood, inflammation, or gut function. A full list of exclusion criteria is provided in the Supplementary Note 2.

### Randomisation and masking

Participant randomisation was performed using the online randomisation list generator provided by Sealed Envelope^58^. A block randomisation method was employed with a fixed block size of 30 to ensure balanced allocation across treatment arms. Participants were randomly assigned 1:1 to one of two groups: exercise or control. Stratified randomisation was applied using three factors to control for potential confounders: age (45–54, 55–65), education level (Secondary, Bachelor, Masters, Doctorate, Other), and sex (Male, Female). A random seed (123456789) was specified to allow reproducibility of the allocation sequence. Given the nature of the trial, participants and researchers were not masked to treatment allocation.

### Procedure

Prior to the baseline visit, participants were asked to fast, refrain from alcohol and avoid strenuous exercise for at least 12 h. At the baseline visit, blood pressure and anthropometric measurements were recorded, followed by self-administered questionnaires; IPAQ, patient health questionnaire (PHQ-9)^36^, 12-item Short Form Health Survey (SF-12)^59^, and the Pittsburgh Sleep Quality Index (PSQI)^60^. Following this, the MST^57^ and Montreal Cognitive Assessment (MoCA)^61^ tasks were administered. Participants then completed a standardised 3-minute step test, after which heart rate was measured over the first minute of recovery. Afterwards, participants rested for 5 min, and their blood sample was collected. Finally, participants were provided with food diaries and stool collection kit and instructed to return a fresh sample within 7 days. Participants were subsequently informed of their assigned group. Those in the exercise group were provided with wearables. Upon study completion, all participants received £100 and travel reimbursement of up to £10.

The exercise programme was co-developed using data from qualitative semi-structured interviews with 9 comparable participants, who fulfilled the study inclusion criteria. Many expressed a preference for a varied format rather than programmes limited to running or aerobic exercise, while emphasising the importance of time efficiency and remote accessibility (Supplementary Table 5; example of participant’s motivation and general opinion around an exercise programme). Participants in the exercise group completed 30-minute group-based online exercise classes three times per week over a 12-week period. Each week included one high-intensity aerobic session (CardioFit), one resistance training session (StrongFit), and one session combining aerobic and static resistance exercises (FunctionalFit). All sessions were delivered using a tabata-style high intensity interval training method. Further details of the exercise programme are provided in Supplementary Table 6. Participants in the control group were instructed to continue with their usual daily routines. Upon completion of the 12-week program, participants attended an endpoint visit, during which the same measures as at baseline were collected. Participants were also offered to attend an optional visit at week 24.

### Outcomes

We measured outcomes at baseline, endpoint (week 12) and an optional follow up visit at week 24. Owing to the limited number of participants who attended the follow-up visit, analyses were restricted to data collected at baseline and endpoint. The primary outcome was pattern separation, assessed using the lure discrimination index (LDI) derived from the MST computerised test^57^. Briefly, the MST comprises two phases: encoding and recognition. During the encoding phase, participants classify 64 images as indoor or outdoor (2 s each, 0.5 s interstimulus interval). Immediately after, they complete the recognition phase with 192 images: exact repeats (targets), novel images (foils), and perceptually similar variants of studied images (lures). Participants then judge these as old, similar or new. LDI is quantified by measuring the rate of “similar” responses given to the lure items, minus “similar” responses given to foils^57^.

Secondary outcomes encompassed measures of cognition, overall well-being, clinical physiology, and blood-based markers. Secondary cognition and overall well-being outcomes, included recognition memory (REC; how well a person recognises old items), measured using the MST^57^; global cognitive function assessed with the MoCA^61^; mental and physical well-being assessed using the mental (MCS-12) and physical (PCS-12) component scores of the SF-12^59^; depressive symptom severity measured with the PHQ-9^36^ and sleep quality, assessed with the PSQI^60^. Secondary clinical outcomes, included cardiovascular fitness, assessed using heart rate (bpm) recorded during the first minute following completion of the 3-minute step test, alongside systolic and diastolic blood pressure, body weight (Kg), waist-to-hip ratio, BMI, and body fat percentage, obtained using a segmental body composition scale. In light of prior evidence linking specific circulating factors to exercise and cognition, secondary blood-based outcomes included serum levels of brain derived neurotrophic factor (BDNF)^21^ and Klotho^62^, as well as plasma levels of Clusterin^21^, quantified using the enzyme-linked immunosorbent assays (ELISAs).

Our primary outcome, pattern separation, is highly dependent on hippocampal neurogenesis^13^, which cannot be measured non-invasively in humans. Therefore, by using an established in vitro parabiosis neurogenesis assay^63,64^, we examined the effect of serum collected from the participants in both exercise and control groups on the expression of neurogenesis-associated markers, in human hippocampal progenitor cells *HPC0A07/03* (HPC; ReNeuron Ltd, UK), in vitro. This assay comprises two main phases, a proliferation and a differentiation phase, and is grounded in evidence that the neurogenic niche directly communicates with the systemic circulation via its vascular interface^65^. It further builds on heterochronic parabiosis studies^39^ and ex vivo serum transfer paradigms^20^ demonstrating that blood-borne factors can alter neurogenesis and hippocampal dependant cognition^51^. Details regarding the assay and the experimental set up can be found in Supplementary Fig. 4 and Supplementary Table 7.

Other prespecified secondary outcomes including gut microbiota composition, serum and stool metabolomics and nutritional intake were assessed only in the exercise subgroup, in accordance with the trial protocol and will be reported in future publications. Individuals were monitored for any adverse events during exercise training and throughout the trial.

### Statistical analysis

The sample size calculations were based on the primary outcome, pattern separation (LDI score). This was informed by data from a published intervention study^66^ which reported a significant positive effect on pattern separation using the MST. Assuming an anticipated effect size of *d* = 0.8 (a large effect size) for the impact of the exercise intervention on pattern separation, with a significance level set at *p* < 0.05 and statistical power at 80%, the minimum required sample size was calculated to be 52 participants. To account for an estimated 15% dropout rate, the recruitment target was set to total of 60 participants.

All statistical analyses were performed using RStudio (Version: 2025.05.1 + 513), in accordance with the CONSORT statement for randomised trials and primarily followed the intention-to-treat principle, inducing all randomised participants with no missing data imputation. In addition to the intention-to-treat analysis, primary and secondary outcomes were also analysed in the per-protocol population. The per-protocol population comprised participants who completed both baseline and endpoint assessments within the protocol-specified visit windows and who met adherence criteria for the intervention. Participants who did not attend the endpoint visit or whose endpoint assessments were conducted outside the allowable window (+ 1 week from the scheduled endpoint) were excluded from the per-protocol analysis. Minor deviations in the timing of baseline assessments (e.g. measures collected later than the prespecified one-week window) were tolerated, provided that the assessments were completed before the start of the intervention. Such deviations were recorded as protocol deviations but were not considered sufficient grounds for exclusion from the per-protocol set.

Changes in outcomes over time between exercise and control groups (intervention * time) were examined using linear mixed-effects models (LMMs; nlme package) and generalised linear mixed-effects models (GLMMs) with a beta distribution (glmmTMB package), selected according to the distributional characteristics of each outcome. Proportion-derived and bounded asymmetric variables were modelled using beta regression, whereas approximately symmetric continuous measures were analysed using Gaussian mixed-effects models. Where required, variables were rescaled to the open unit interval to satisfy distributional assumptions of the beta family. Model assumptions were evaluated using residual diagnostics appropriate to each modelling framework. Both models included fixed effects for intervention group, time, their interaction and were adjusted for age, sex, years of education (≤ 12 vs >12), and baseline values of the outcome. A random intercept was included for each participant to account for within-subject correlations due to repeated measurements. Ordinal IPAQ categories were modelled using a cumulative link mixed-effects model (CLMM) with a logit link. Fixed effects included intervention group, time and their interaction, with sex, age, and years of education included as covariates. A random intercept for participants accounted for within-participant clustering across repeated measurements. Separate models were constructed for each outcome. Statistical significance was set at *p* < 0.05. No adjustments for multiple testing were applied to the primary outcome; however, secondary outcomes were adjusted for multiple comparisons using the Benjamini–Hochberg false discovery rate (FDR) method.

## Supplementary information

Supplementary information

## Data Availability

The de-identified data supporting this article is available under a Data Access Agreement from the King’s College London research data repository, KORDS, at https://doi.org/10.18742/32642109.

## Code availability

Representative R Markdown scripts illustrating the statistical analyses performed in this study are publicly available at: https://github.com/thuretlabkcl/NeuroFit-RCT-Statistical-Code.git.
