# Supplementary material for: Effects of a three-month exercise programme on cognition, mood and neurogenesis: the NeuroFit randomised controlled trial
Source: NPJ Aging. 2026 Sep 17;12(1):124. doi: 10.1038/s41514-026-00439-w (PMC13586222; doi:10.1038/s41514-026-00439-w)
Supplement: Supplementary file 1 [file 41514_2026_439_MOESM1_ESM.pdf]

Supplementary information

Effects of a three-month exercise programme on cognition, mood and neurogenesis: The NeuroFit randomised controlled trial

Authors

Sahand Farmand[a], Curie Kim[a], Andrea Du Preez[a], André O. Werneck[b], Lucia Batzu[a], Dominic Pearce[b], Kirsten Brady[a], Markos Khir[a], Sarah Nicolas[c,d], John F. Cryan[c,d], Yvonne M. Nolan[c,d], Brendon Stubbs[b], Sandrine Thuret*[a]

Affiliations

a Department of Basic and Clinical Neuroscience, Institute of Psychiatry, Psychology and Neuroscience, King's College London, London, United Kingdom.

b Department of Psychological Medicine, Institute of Psychiatry, Psychology and Neuroscience, King's College London, London, United Kingdom.

c Department of Anatomy and Neuroscience, University College Cork, Cork, Ireland.

d APC Microbiome Ireland, University College Cork, Cork, Ireland.

Supplementary Table 1. Indicates the results (estimates (95% CI), standard error, *p* value and the adjusted *p* value), for intervention by time for select clinical and blood-based outcomes, for the per-protocol analysis. The interaction term (*) represents the difference in change over time between the exercise and control groups and has been adjusted for age, sex, years of education and baseline differences. The *p*-value for the primary outcome has not been adjusted, whereas for the secondary outcomes, it has been adjusted using the Benjamini–Hochberg false discovery rate method. (LDI; Lure discrimination index, REC; Recognition memory, MoCA; Montreal Cognitive assessment, PHQ-9; Patient health questionnaire-9, MCS12; Mental component of short form questionnaire-12, PCS12; Physical component of short form questionnaire, PSQI; Pittsburgh sleep quality index, HR; Heart rate, BMI; Body mass index, BP; Blood pressure).

**Per-protocol**		**Intervention*time**			
**Outcomes**	***n***	**Estimate (95% CI)**	**Standard error**	***p***	**Adjusted *p***
**Primary outcome**					
LDI	35	0.27 (-0.46; 1.01)	0.37	0.47	-
**Secondary outcomes**					
REC	35	0.33 (-0.41; 1.11)	0.38	0.38	0.60
MoCA	35	-0.010 (-1.30, 1.32)	0.64	0.99	0.99
PHQ-9	35	-1.21 (-2.14, -0.27)	0.48	0.012	0.047
MCS12	35	-0.27 (-0.88; 0.33)	0.31	0.38	0.38
PCS12	35	0.22 (-0.45; 0.90)	0.35	0.52	0.93
PSQI	35	0.063 (-1.80; 1.92)	0.91	0.94	0.94
Resting HR (BPM)	35	0.50 (-5.45; 6.44)	2.92	0.87	0.87
HR (BPM) post 3’ step test	35	2.64 (-2.72; 8.00)	2.63	0.32	0.43
Weight	35	-0.37 (-1.75; 1.01)	0.68	0.59	0.77
Waist to hip ratio	35	0.0098 (-0.029; 0.049)	0.019	0.61	0.79
BMI	35	-0.14 (-0.62; 0.35)	0.24	0.57	0.91
Body fat (%)	35	0.073 (-0.0057; 0.15)	0.040	0.069	0.092
Systolic BP	35	-0.64 (-7.90; 6.62)	3.57	0.86	0.93
Diastolic BP	35	2.64 (-2.34; 7.13)	2.27	0.25	0.41
Serum BDNF (ng/mL)	34	0.93 (-2.56; 4.43)	1.72	0.59	0.94
Serum Klotho (ng/mL)	35	0.016 (-0.42; 0.46)	0.22	0.94	0.94
Plasma Clusterin (μg/mL)	32	7.33 (-6.06; 20.72)	6.56	0.27	0.60

Supplementary Table 2. Indicates the results (estimates (95% CI), standard error, *p* value and the adjusted *p* value), for intervention by time for secondary neurogenesis outcomes during proliferation and differentiation phase, for the per-protocol analysis. The interaction term (*) represents the difference in change over time between the exercise and control groups and has been adjusted for baseline differences and immunostaining batch. The *p*-value for the secondary neurogenesis outcomes has been adjusted using the Benjamini–Hochberg false discovery rate method. (Ki67; Kiel-67 antigen; cell proliferation marker, CC3; Cleaved Caspase-3; apoptotic marker, Nestin; neuroepithelial stem cell protein; progenitor integrity marker, Sox2; SRY-box transcription factor 2; stem cell ness marker, DCX; Doublecortin; immature neurons marker, MAP2; Microtubule-Associated Protein 2; mature neurons marker).

**Per-protocol**		**Intervention * time**			
**Secondary neurogenesis outcomes**	***n***	**Estimate (95% CI)**	**Standard error**	***p***	**Adjusted *p***
**Proliferation phase**					
%Ki67 positive cells	34	-0.26 (-0.46; -0.053)	0.10	0.013	**0.027**
%CC3 positive cells	34	0.035 (-0.096; 0.17)	0.067	0.60	0.69
%Nestin positive cells	34	-0.33 (-0.64; -0.020)	0.16	0.037	0.098
%Sox2 positive cells	34	-0.38 (-1.17; 0.42)	0.41	0.36	0.41
Cell number	34	99.66 (-291.10; 490.40)	191.84	0.61	0.93
**Differentiation phase**					
%Ki67 positive cells	34	0.0059 (-0.059; 0.071)	0.033	0.86	0.98
%CC3 positive cells	34	0.020 (-0.12; 0.16)	0.069	0.77	0.77
%DCX positive cells	34	-0.027 (-0.19; 0.14)	0.084	0.75	0.86
%MAP2 positive cells	34	0.051 (-0.10; 0.21)	0.079	0.52	0.70
Neurite length (px)	34	-0.10 (-0.31; 0.11)	0.10	0.32	0.37
Cell number	34	10.71 (-191.63; 213.10)	99.37	0.91	0.91

Supplementary Table 3. Unadjusted baseline and endpoint descriptive statistics for clinical, cognitive, and physiological outcomes in the exercise and control groups, stratified by sex (Intention-to-treat population).

**Exercise group**			
**Outcome**	**Sex**	**Baseline, n, mean (SD)**	**Endpoint, n, mean (SD)**
Body fat (%)	Female	n=20; 32.00 (5.00)	n=19; 33.00 (6.00)
Body fat (%)	Male	n=7; 24.00 (5.00)	n=6; 25 (3.00)
BMI	Female	n=20; 24.99 (2.79)	n=19; 24.97 (2.74)
BMI	Male	n=7; 26.52 (2.11)	n=6; 26.54 (0.98)
Diastolic BP	Female	n=20; 74.95 (9.22)	n=19; 75.86 (8.88)
Diastolic BP	Male	n=7; 75.86 (9.84)	n=6; 74.56 (9.96)
HR (BPM) Post 3' step test	Female	n=20; 73.70 (8.69)	n=19; 73.16 (11.79)
HR (BPM) Post 3' step test	Male	n=7; 69.00 (5.23)	n=6; 73.83 (11.41)
Resting HR (BPM)	Female	n=20; 67.43 (8.08)	n=19; 66.71 (11.75)
Resting HR (BPM)	Male	n=7; 65.05 (4.96)	n=6; 66.42 (9.92)
LDI	Female	n=20; 0.20 (0.19)	n=19; 0.26 (0.17)
LDI	Male	n=7; 0.24 (0.16)	n=6; 0.34 (0.16)
MCS12	Female	n=20; 53.54 (6.21)	n=19; 55.31 (3.36)
MCS12	Male	n=7; 53.54 (9.12)	n=6; 51.80 (9.13)
MoCA	Female	n=20; 24.60 (2.93)	n=19; 25.47 (2.57)
MoCA	Male	n=7; 25.14 (2.12)	n=6; 25.67 (1.63)
PCS12	Female	n=20; 52.75 (7.02)	n=19; 52.65 (4.29)
PCS12	Male	n=7; 53.78 (3.24)	n=6; 55.59 (1.95)
PHQ-9	Female	n=20; 2.85 (2.32)	n=19; 1.84 (2.24)
PHQ-9	Male	n=7; 2.57 (4.69)	n=6; 2.00 (3.46)
PSIQ	Female	n=17; 3.76 (1.68)	n=19; 4.21 (2.10)
PSIQ	Male	n=5; 3.60 (1.14)	n=6; 5.50 (2.74)
REC	Female	n=20; 0.71 (0.23)	n=19; 0.71 (0.20)
REC	Male	n=7; 0.80 (0.06)	n=6; 0.75 (0.08)
Systolic BP	Female	n=20; 116.38 (13.05)	n=19; 116.32 (8.74)

Systolic BP	Male	n=7; 117.48 (15.89)	n=6; 118.94 (11.22)
Weight (Kg)	Female	n=20; 65.23 (8.48)	n=19; 65.28 (8.68)
Weight (Kg)	Male	n=7; 84.69 (8.46)	n=6; 84.25 (7.20)
Waist to hip ratio	Female	n=20; 0.85 (0.05)	n=19; 0.86 (0.06)
Waist to hip ratio	Male	n=7; 0.92 (0.08)	n=6; 0.90 (0.03)

**Control group**			
**Outcome**	**Sex**	**Baseline, n, mean (SD)**	**Endpoint, n, mean (SD)**
Body fat (%)	Female	n=17; 32.00 (6.00)	n=14; 32.00 (5.00)
Body fat (%)	Male	n=8; 24.00 (5.00)	n=8; 21.00 (5.00)
BMI	Female	n=17; 24.22 (2.66)	n=14; 24.42 (2.70)
BMI	Male	n=8; 26.31 (2.91)	n=8; 25.93 (2.56)
Diastolic BP	Female	n=17; 79.86 (11.18)	n=14; 79.30 (7.95)
Diastolic BP	Male	n=8; 83.62 (10.27)	n=8; 77.75 (5.90)
HR (BPM) Post 3' step test	Female	n=17; 79.00 (12.40)	n=14; 76.43 (10.14)
HR (BPM) Post 3' step test	Male	n=8; 73.88 (8.74)	n=8; 65.62 (8.25)
Resting HR (BPM)	Female	n=17; 70.06 (9.54)	n=14; 71.73 (10.76)
Resting HR (BPM)	Male	n=8; 69.21 (9.71)	n=8; 64.48 (8.92)
LDI	Female	n=17; 0.32 (0.21)	n=14; 0.34 (0.18)
LDI	Male	n=8; 0.27 (0.11)	n=8; 0.24 (0.16)
MCS12	Female	n=17; 49.87 (10.04)	n=14; 50.99 (8.51)
MCS12	Male	n=8; 55.34 (2.88)	n=8; 55.08 (2.74)
MOCA	Female	n=17; 25.35 (2.15)	n=14; 26.43 (1.83)
MOCA	Male	n=8; 25.50 (2.14)	n=8; 26.12 (1.96)
PCS12	Female	n=17; 52.01 (4.42)	n=14; 51.83 (6.04)
PCS12	Male	n=8; 52.69 (5.19)	n=8; 50.61 (7.35)
PHQ-9	Female	n=17; 3.76 (2.49)	n=14; 4.29 (3.93)
PHQ-9	Male	n=8; 1.00 (1.07)	n=8; 1.50 (1.31)
PSIQ	Female	n=16; 5.88 (1.89)	n=14; 7.07 (3.63)
PSIQ	Male	n=5; 4.00 (0.71)	n=8; 4.88 (1.46)

REC	Female	n=17; 0.70 (0.21)	n=14; 0.77 (0.12)
REC	Male	n=8; 0.81 (0.13)	n=8; 0.70 (0.25)
Systolic BP	Female	n=17; 120.57 (16.97)	n=14; 123.69 (13.15)
Systolic BP	Male	n=8; 128.04 (13.29)	n=8; 123.81 (15.17)
Weight (Kg)	Female	n=17; 62.67 (8.93)	n=14; 62.64 (7.92)
Weight (Kg)	Male	n=8; 82.41 (14.03)	n=8; 81.20 (13.10)
Waist to hip ratio	Female	n=17; 0.84 (0.06)	n=14; 0.85 (0.05)
Waist to hip ratio	Male	n=8; 0.95 (0.03)	n=8; 0.91 (0.09)

Supplementary Table 4. Estimated marginal means and 95% confidence intervals for the primary, secondary well-being, clinical and neurogenesis outcomes by intervention group and time point. Estimates were derived from linear mixed-effects models including fixed effects for intervention group, time, and their interaction, and adjusted for age, sex, years of education, and baseline values, following an intention-to-treat approach. Model-based estimated marginal means were obtained on the normalized scale and subsequently back-transformed to the original scale for interpretability. Back-transformed means and confidence intervals are presented for baseline and endpoint assessments (intention-to-treat population).

**Outcomes**	**Control group (n=25)**	**Exercise group (n=27)**
	**Estimated marginal mean [95% CI]**	**Estimated marginal mean [95% CI]**
**Primary outcome**		
**LDI**		
Baseline	0.22 [0.16; 0.28]	0.25 [0.19; 0.31]
Endpoint	0.27 [0.19; 0.33]	0.35 [029; 0.41]
**Secondary clinical outcomes**		
**REC**		
Baseline	0.77 [0.71; 0.81]	0.72 [0.66; 0.77]
Endpoint	0.75 [0.67; 0.80]	0.73 [0.66; 0.79]
**MoCA**		
Baseline	25.09 [24.36; 25.81]	24.87 [24.18; 25.56]
Endpoint	26.03 [25.28; 26.79]	25.73 [25.02; 26.43]
**PHQ-9**		
Baseline	1.69 [1.00; 2.78]	1.79 [1.08; 2.86]
Endpoint	2.25 [1.36; 3.56]	0.82 [0.45; 1.46]
**MCS12**		
Baseline	51.60 [49.01, 53.98]	52.16 [49.65, 54.47]
Endpoint	55.39 [52.78, 57.60]	53.57 [50.93, 55.91]
**PCS12**		
Baseline	52.24 [49.95; 54.27]	51.34 [49.10; 53.37]
Endpoint	53.12 [50.81; 55.14]	52.35 [49.99; 54.43]
**PSQI**		
Baseline	4.87 [3.69; 6.10]	3.79 [2.66; 4.91]
Endpoint	5.88 [4.74; 7.01]	4.45 [3.35; 5.54]
**Resting HR (BPM)**		
Baseline	69.78 [64.69; 74.87]	66.94 [62.17; 71.71]
Endpoint	69.88 [64.71; 75.04]	66.76 [61.94; 71.59]
**HR (BPM) post 3' step**		
**test**	74.96 [72.23; 77.68]	74.12 [71.48; 76.76]
Baseline	71.89 [69.09; 74.70]	74.97 [72.27; 77.68]
Endpoint		
**Weight (Kg)**		
Baseline	69.75 [69.10; 70.39]	69.74 [69.10; 70.38]
Endpoint	69.76 [68.91; 70.24]	69.39 [68.73; 70.04]
**Waist to hip ratio**		
Baseline	0.88 [0.86; 0.90]	0.88 [0.86; 0.89]
Endpoint	0.87 [0.85; 0.89]	0.87 [0.85; 0.89]

**BMI**		
Baseline	25.15 [24.93; 25.38]	25.15 [24.93; 25.37]
Endpoint	25.13 [24.90; 25.36]	25.05 [24.82; 25.27]
**Body fat (%)**		
Baseline	29.44 [28.58; 30.31]	29.32 [28.50; 30.17]
Endpoint	28.21 [27.33; 29.19]	29.60 [28.71; 30.40]
**Systolic BP**		
Baseline	120.90 [117.58; 124.22]	119.52 [116.34; 122.69]
Endpoint	122.79 [119.37; 126.20]	118.90 [115.65; 122.15]
**Diastolic BP**		
Baseline	77.92 [75.49; 80.35]	76.59 [74.30; 78.88]
Endpoint	76.19 [73.69; 78.70]	76.20 [73.85; 78.55]
**Serum BDNF (ng/mL)**		
Baseline	10.10 [8.64; 11.57]	9.87 [8.46; 11.28]
Endpoint	10.44 [8.88; 12.01]	11.39 [9.95; 12.84]
**Serum Klotho**		
**(ng/mL)**	3.33 [3.11; 3.55]	3.32 [3.11; 3.54]
Baseline	3.28 [3.06; 3.51]	3.20 [2.98; 3.43]
Endpoint		
**Plasma Clusterin**		
**(µg/mL)**	62.33 [55.32; 69.32]	59.95 [53.53; 66.37]
Baseline	57.77 [50.26; 65.27]	62.39 [55.81; 68.98]
Endpoint		

	**Control group (n=25)**	**Exercise group (n=27)**
**Secondary neurogenesis outcomes**	**Estimated marginal mean [95% CI]**	**Estimated marginal mean [95% CI]**
**Proliferation phase**		
%Ki67 positive cells		
Baseline	96.41 [95.99, 96.78]	96.65 [96.25, 97.01]
Endpoint	96.61 [96.17, 97.00]	96.04 [95.59, 96.45]
%CC3 positive cells		
Baseline	0.92 [0.85, 0.98]	0.92 [0.86, 0.98]
Endpoint	0.83 [0.77, 0.90]	0.86 [0.80, 0.92]
%Nestin positive cells		
Baseline	98.52 [98.31, 98.71]	98.55 [98.34, 98.74]
Endpoint	98.61 [98.38, 98.81]	98.37 [98.13, 98.58]
%SOX2 positive cells		
Baseline	99.87 [99.80, 99.91]	99.90 [99.84, 99.93]
Endpoint	99.88 [99.81, 99.93]	99.87 [99.80, 99.91]
Number of cells		
Baseline	7575 [7409; 7743]	7592 [7429; 7756]
Endpoint	7250 [7059; 7441]	7312 [7143; 7482]
**Differentiation phase**		
%Ki67 positive cells		
Baseline	59.28 [58.58, 59.97]	59.08 [58.39, 59.76]
Endpoint	58.65 [57.86, 59.44]	58.64 [57.92, 59.36]

%CC3 positive cells<br>Baseline<br>Endpoint	 5.40 [5.04; 5.77]<br>6.39 [5.93; 6.88]	 5.21 [4.86; 5.59]<br>6.20 [5.79; 6.63]
%MAP2 positive cells<br>Baseline<br>Endpoint	 89.77 [88.62; 90.82]<br>88.42 [87.08; 89.64]	 89.25 [88.06; 90.34]<br>87.24 [85.87; 88.50]
%DCX positive cells<br>Baseline<br>Endpoint	 93.07 [92.33; 93.74]<br>92.79 [91.95; 93.55]	 93.04 [92.30; 93.71]<br>91.53 [90.65; 92.32]
Number of cells<br>Baseline<br>Endpoint	 9736 [9598; 9875]<br>9584 [9427; 9743]	 9683 [9557; 9820]<br>9422 [9281; 9564]
Mean neurite length (px)<br>Baseline<br>Endpoint	 16.31 [16.15; 16.41]<br>16.57 [16.41; 16.74]	 16.36 [16.22; 16.50]<br>16.61 [16.47; 16.76]

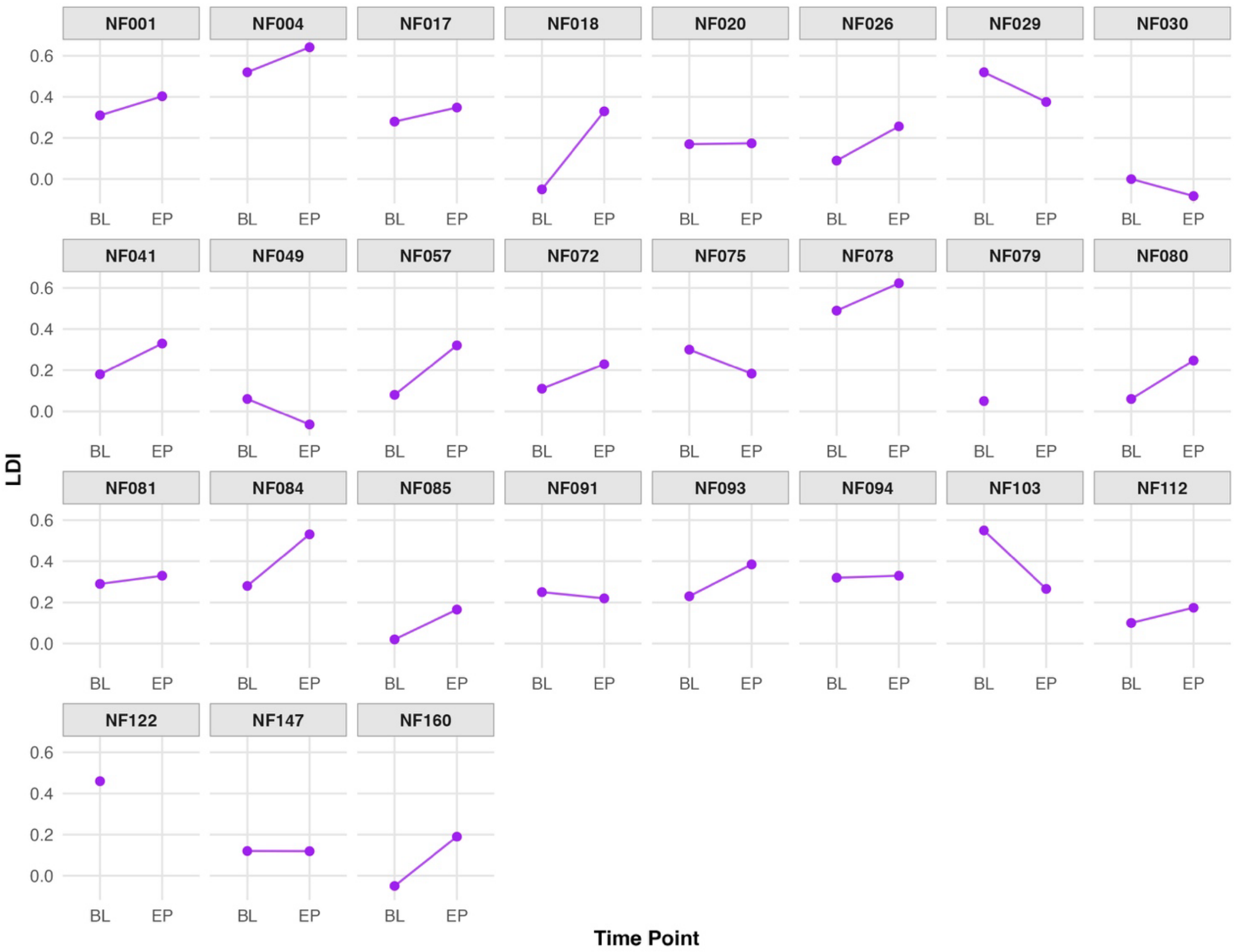

Supplementary Figure 1. Change in LDI across baseline (BL) and endpoint (EP) for each participant in the exercise intervention group. Each panel represents an individual participant, with lines connecting within-participant values. This faceted spaghetti plot illustrates individual variability in baseline levels and response trajectories, highlighting heterogeneity in exercise-related changes. 'NF' indicates NeuroFit, and the number that follows corresponds to the participant ID.

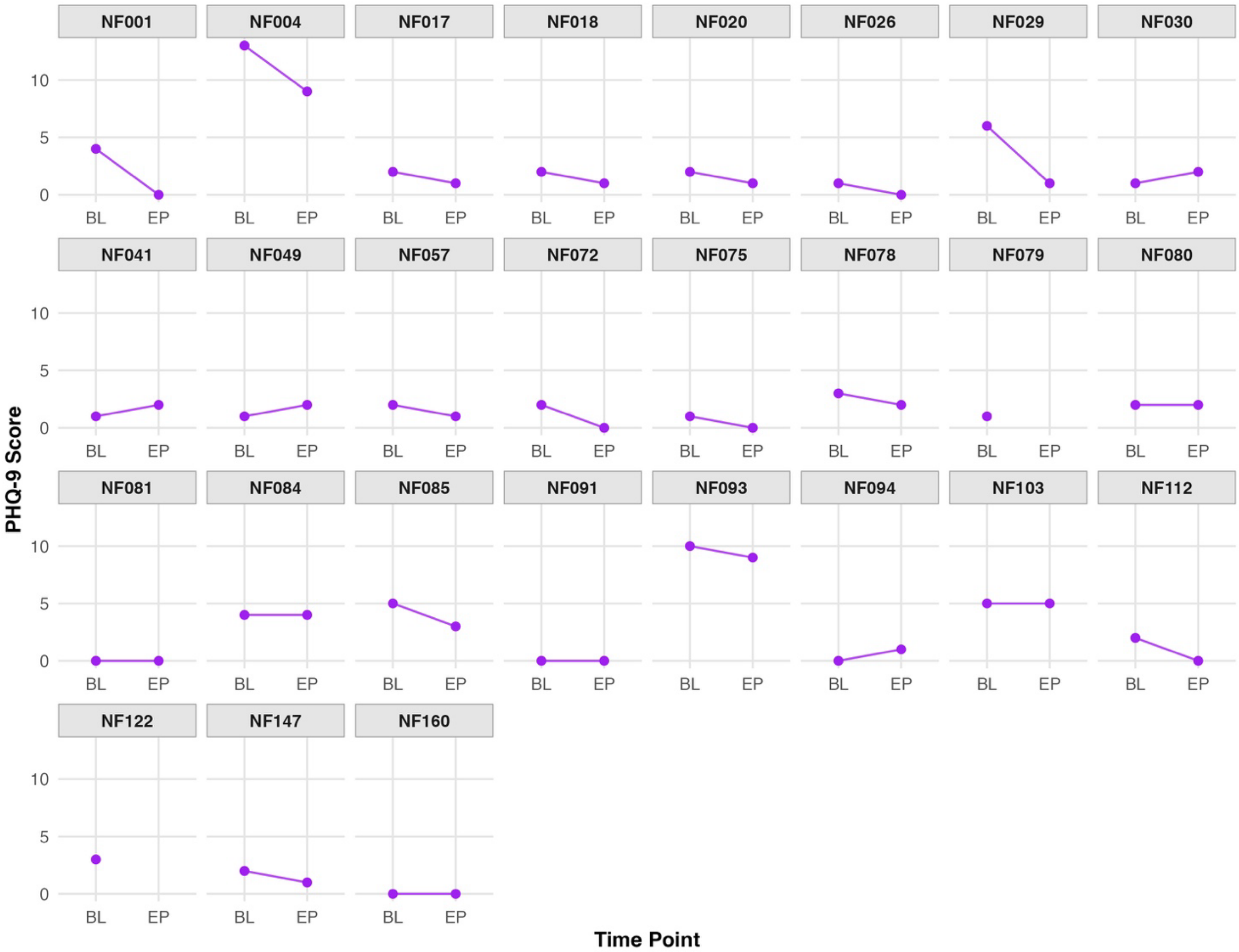

Supplementary Figure 2. Change in PHQ-9 score across baseline (BL) and endpoint (EP) for each participant in the exercise intervention group. Each panel represents an individual participant, with lines connecting within-participant values. This faceted spaghetti plot illustrates individual variability in baseline levels and response trajectories, highlighting heterogeneity in exercise-related changes. 'NF' indicates NeuroFit, and the number that follows corresponds to the participant ID.

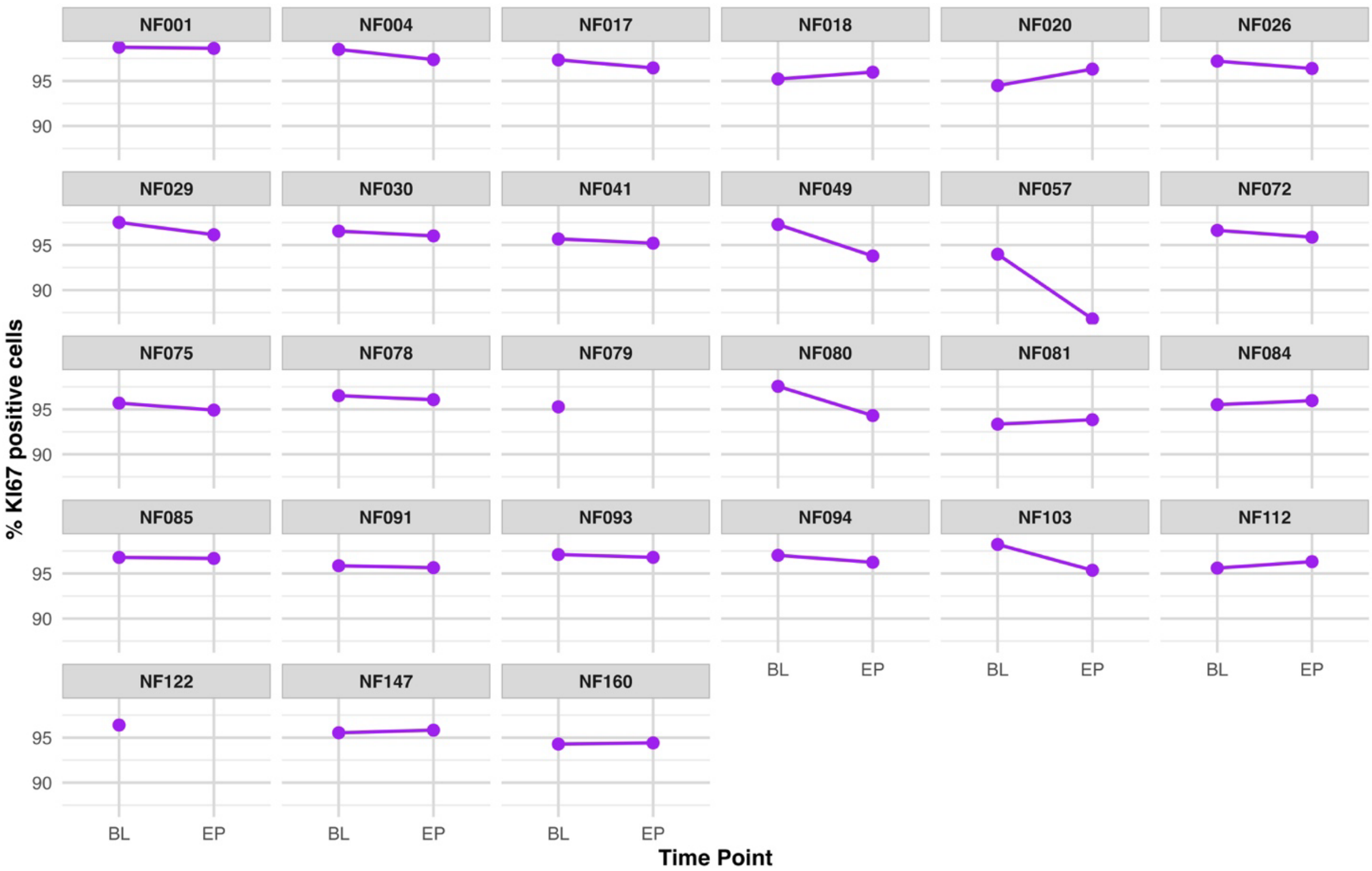

Supplementary Figure 3. Change in % Ki67 positive cells across baseline (BL) and endpoint (EP) for each participant in the exercise intervention group. Each panel represents an individual participant, with lines connecting within-participant values. This faceted spaghetti plot illustrates individual variability in baseline levels and response trajectories, highlighting heterogeneity in exercise-related changes. 'NF' indicates NeuroFit, and the number that follows corresponds to the participant ID.

Supplementary Note 1. Blood Collection and Processing

Materials

8.5 mL serum separator tube (SST; yellow), 4 mL fluoride oxalate tube (grey), 4 mL EDTA tube (purple), along with a tourniquet, alcohol wipe, butterfly needle, cotton, tape, pillow, and gloves.

Screening visit

At the screening visit, venous blood was collected by a trained phlebotomist using standard aseptic technique. After collection, tubes were gently inverted 5–8 times. The SST was allowed to stand at room temperature for at least 10 minutes to permit clotting, while other tubes were stored refrigerated until processing. Tubes, excluding the EDTA, were centrifuged at 1500 × g for 15 minutes at 4 °C. Serum and plasma were aliquoted into pre-labelled Eppendorf tubes according to analyte requirements. Aliquots were placed in padded envelopes, sealed with biohazard tape, and prepared for shipping under controlled conditions.

Study visit

At each study visit, blood was collected following the same procedures using two 8.5 mL SST tubes (yellow) and one 4 mL fluoride oxalate tube (grey). After collection and inversion, SST tubes were left at room temperature for at least 10 minutes to clot, while other tubes were kept refrigerated. Tubes were centrifuged at 1500 × g for 15 minutes at 4 °C. Serum was aliquoted into pre-labelled Eppendorf tubes, with remaining spare serum retained for future analyses.

Supplementary Note 2. Full exclusion criteria

Participants were excluded if they (1) could not understand the information sheet or (2) provide informed consent, (3) were unable to complete cognitive testing, or (4) had uncorrected vision impairments, who (5) smoked, (6) would not maintain their habitual diet, (7) refused blood or stool sampling, (8) were not in general good health, (9) could not engage in structured exercise per the physical activity readiness questionnaire (PARQ), or (10) were unwilling to exercise three times weekly. Individuals who (11) were pregnant, lactating, or planning pregnancy were also excluded. Mental-health exclusions covered (12) recent (past 2 years) or current psychiatric diagnoses (e.g., depression, anxiety, bipolar disorder, schizophrenia). Medical exclusions included (13) significant cardiovascular, respiratory, gastrointestinal, or liver disease, (14) major gastrointestinal surgery (except appendectomy or cholecystectomy), (15) cancer within 5 years (excluding melanoma), and (16) abnormal screening blood results. Exclusions applied to those with (17) functional gastrointestinal disorders (e.g., inflammatory bowel disease (IBD), coeliac disease, irritable bowel syndrome (IBS)), (18) functional diarrhoea or constipation (Rome IV), (19) neurological disease (e.g., Alzheimer's, Parkinson's, stroke), (20) neurodevelopmental disorders affecting cognition, or (21) traumatic brain injury or other cognitive-impairing conditions. Additional criteria excluded participants with (22) uncontrolled epilepsy or fainting tendency, (23) eating disorders, (24) metabolic conditions (type 1 or 2 diabetes), (25) unwillingness to stop pre/probiotics ≥4 weeks pre-study, or (26) alcohol misuse (>14 drinks/week). Exclusions applied to those who (27) had sleep disorders or irregular overnight work, (28) routinely used specified medications (psychoactive, laxatives, antibiotics, nonsteroidal anti-inflammatory drugs (NSAIDs), anticoagulants, proton pump inhibitors (PPIs)) and were unwilling to pause them, or (29) used cognition-related medications (statins, metformin, Alzheimer's drugs). Finally, (30) any condition judged by researchers to compromise consent, compliance, safety, or data integrity led to exclusion.

Supplementary Table 5. Example of participant's motivation and general attitude around exercise which was used to co-design the exercise programme. (Obtained from qualitative semi-structured interviews conducted with a separate group of individuals matched for characteristics to the study cohort).

**General Attitudes Around Exercise**			
**Difficulties in habit building and self-motivation**	**Health and fitness priority overweight loss**	**Difficulties in time management**	**Motivated by social circles**
"I wish I was more motivated. I don't really get the endorphins that everyone talks about, and I find it difficult to maintain exercise."	*"I don't want to get ill for my kids or, I don't, I don't want to get ill for me. I want to live a long and healthy life. "*	*"So, I think the hardest thing for me is just finding the time you know between working pretty much full time, having two relatively small children and a dog. "*	*"I did have a very rare opportunity to spend time outside exercising with friends… I found that to be much more engaging."*

**Motivation/Compliance to The Study**			
**Practicality – time and location**	**Intensity to be scaled to the individual**	**Forming social bonds**	**Reinforce that they must attend**
"I wouldn't travel, you know, wouldn't travel more than half an hour to go to a gym."	*"I'd like to get to a point where I'd happily get sweaty and out of breath, but I don't think that realistically… It would… If I'm realistic it would probably it put me off. If it went, went straight in."*	*"Getting to know other people in the group and having a sort of sense of 'we're doing this together'"*<br><br>*"I think it can be made fun and that depends on the*	*"You know there isn't oh you can come, or you can't come. It's like you come or you're out of the program."*

		facilitators and the instructors and the group that're doing it with us I suppose. "	

Supplementary Table 6. Details of the exercise programme (EMOM = every minute on the minute)

	**CardioFit**	**StrongFit**	**FunctionalFit**
**Week 1**	30-minute circuit<br><br>**6 rounds**<br><br>**2 movements: 1 minute work, 90s rest**<br><br>Movement 1 – Star jumps<br>Movement 2 – High knees<br><br>Movements can be replaced with others depending on participant's ability and injuries. Examples include marches, mountain climbers, step ups.	30-minute circuit: 40 seconds work, 20 seconds rest using dumbbells or kettlebells or resistance bands.<br><br>1-minute rest between rounds<br><br>**Lower body – EMOM 3 rounds**<br>Min 1 – Squats (modifications: chair sits)<br>Min 2 – Romanian deadlift<br>Min 3 – Forward lunge (modification: shorter stride)<br>Min 4 – Standing calf raises (modification: seated)<br>Min 5 – Lying glute bridge<br><br>**Upper body & core – EMOM 3 rounds**<br>Min 1 – Press ups (modifications: wall, raised, knees)<br>Min 2 – Single arm bent over rows<br>Min 3 – Shoulder press<br>Min 4 – Bird dog<br>Min 5 – Dead bug	30-minute circuit: 40 seconds work, 20 seconds rest alternating cardio and static resistance.<br><br>1-minute rest between each round<br><br>**5 rounds**<br><br>Station 1 – Air punches<br>Station 2 – Plank (modifications: on hands, raised, on knees)<br>Station 3 – Burpees (modifications: no jump, down onto knees and step feet back, down to plank position rather than chest to floor)<br>Station 4 – Farmers hold<br>Station 5 – Step ups<br>Station 6 – Wall sit (modification: angle of knee bend)
**Week 2**	30-minute circuit<br><br>**6 rounds**<br><br>**2 movements: 1 minute work, 90s rest**<br>Movement 1 – Star jumps<br>Movement 2 – High knees	30-minute circuit: 40 seconds work, 20 seconds rest using dumbbells or kettlebells or resistance bands.<br><br>1-minute rest between rounds<br><br>**Lower body – EMOM 3 rounds**<br>Min 1 – Squats (modifications: chair sits)<br>Min 2 – Romanian deadlift	30-minute circuit: 40 seconds work, 20 seconds rest alternating cardio and static resistance.<br><br>1-minute rest between each round<br><br>**5 rounds**<br><br>Station 1 – Squat jumps (modification: no jump)<br>Station 2 – Hollow hold

	Movements can be replaced with others depending on participant's ability and injuries. Examples include marches, mountain climbers, step ups.	Min 3 – Forward lunge (modification: shorter stride)<br>Min 4 – Standing calf raises (modification: seated)<br>Min 5 – Lying glute bridge<br><br>**Upper body & core – EMOM 3 rounds**<br>Min 1 – Press ups (modifications: wall, raised, knees)<br>Min 2 – Single arm bent over rows<br>Min 3 – Shoulder press<br>Min 4 – Bird dog<br>Min 5 – Dead bug	Station 3 – Mountain climbers (modification: raised)<br>Station 4 – Balance on one leg, change side halfway (easy: keep raised leg low, intermediate: have knee up at 90-degree angle, advanced: slight bend in standing leg)<br>Station 5 – Marches<br>Station 6 – Chair pose (modification: angle of knee bend)

	**CardioFit**	**StrongFit**	**FunctionalFit**
**Week 3**	30-minute circuit<br><br>**6 rounds**<br><br>**2 movements: 1 minute work, 90s rest**<br>Movement 1 – Star jumps<br>Movement 2 – High knees<br><br>Movements can be replaced with others depending on participant's ability and injuries. Examples include marches, mountain climbers, step ups.	30-minute circuit: 40 seconds work, 20 seconds rest using dumbbells or kettlebells or resistance bands.<br><br>1-minute rest between rounds<br><br>**Lower body – EMOM 3 rounds**<br>Min 1 – Squats (modifications: chair sits)<br>Min 2 – Romanian deadlift<br>Min 3 – Forward lunge (modification: shorter stride)<br>Min 4 – Standing calf raises (modification: seated)<br>Min 5 – Lying glute bridge<br><br>**Upper body & core – EMOM 3 rounds**<br>Min 1 – Press ups (modifications: wall, raised, knees)<br>Min 2 – Single arm bent over rows	30-minute circuit: 40 seconds work, 20 seconds rest alternating cardio and static resistance.<br><br>1-minute rest between each round<br><br>**5 rounds**<br><br>Station 1 – Reverse lunges<br>Station 2 – Plank (modifications: on hands, against wall, raised, on knees)<br>Station 3 – Jumping jacks<br>Station 4 – Superman<br>Station 5 – Step ups<br>Station 6 – Bear hold

		Min 3 – Shoulder press<br>Min 4 – Bird dog<br>Min 5 – Dead bug	
**Week 4**	30-minute circuit<br><br>**6 rounds**<br><br>**2 movements: 1 minute work, 90s rest**<br>Movement 1 – Star jumps<br>Movement 2 – High knees<br><br>Movements can be replaced with others depending on participant's ability and injuries. Examples include marches, mountain climbers, step ups.	30-minute circuit: 40 seconds work, 20 seconds rest using dumbbells or kettlebells or resistance bands.<br><br>1-minute rest between rounds<br><br>**Lower body – EMOM 3 rounds**<br>Min 1 – Squats (modifications: chair sits)<br>Min 2 – Romanian deadlift<br>Min 3 – Forward lunge (modification: shorter stride)<br>Min 4 – Standing calf raises (modification: seated)<br>Min 5 – Lying glute bridge<br><br>**Upper body & core – EMOM 3 rounds**<br>Min 1 – Press ups (modifications: wall, raised, knees)<br>Min 2 – Single arm bent over rows<br>Min 3 – Shoulder press<br>Min 4 – Bird dog<br>Min 5 – Dead bug	30-minute circuit: 40 seconds work, 20 seconds rest alternating cardio and static resistance.<br><br>1-minute rest between each round<br><br>**5 rounds**<br><br>Station 1 – Squat jumps (modification: no jump)<br>Station 2 – Farmers hold<br>Station 3 – Walkouts (modification: down onto knees first)<br>Station 4 – Balance on one leg, change side halfway (easy: keep raised leg low, intermediate: have knee up at 90-degree angle, advanced: slight bend in standing leg)<br>Station 5 – Flutter kicks<br>Station 6 – Wall sit (modification: angle of knee bend)

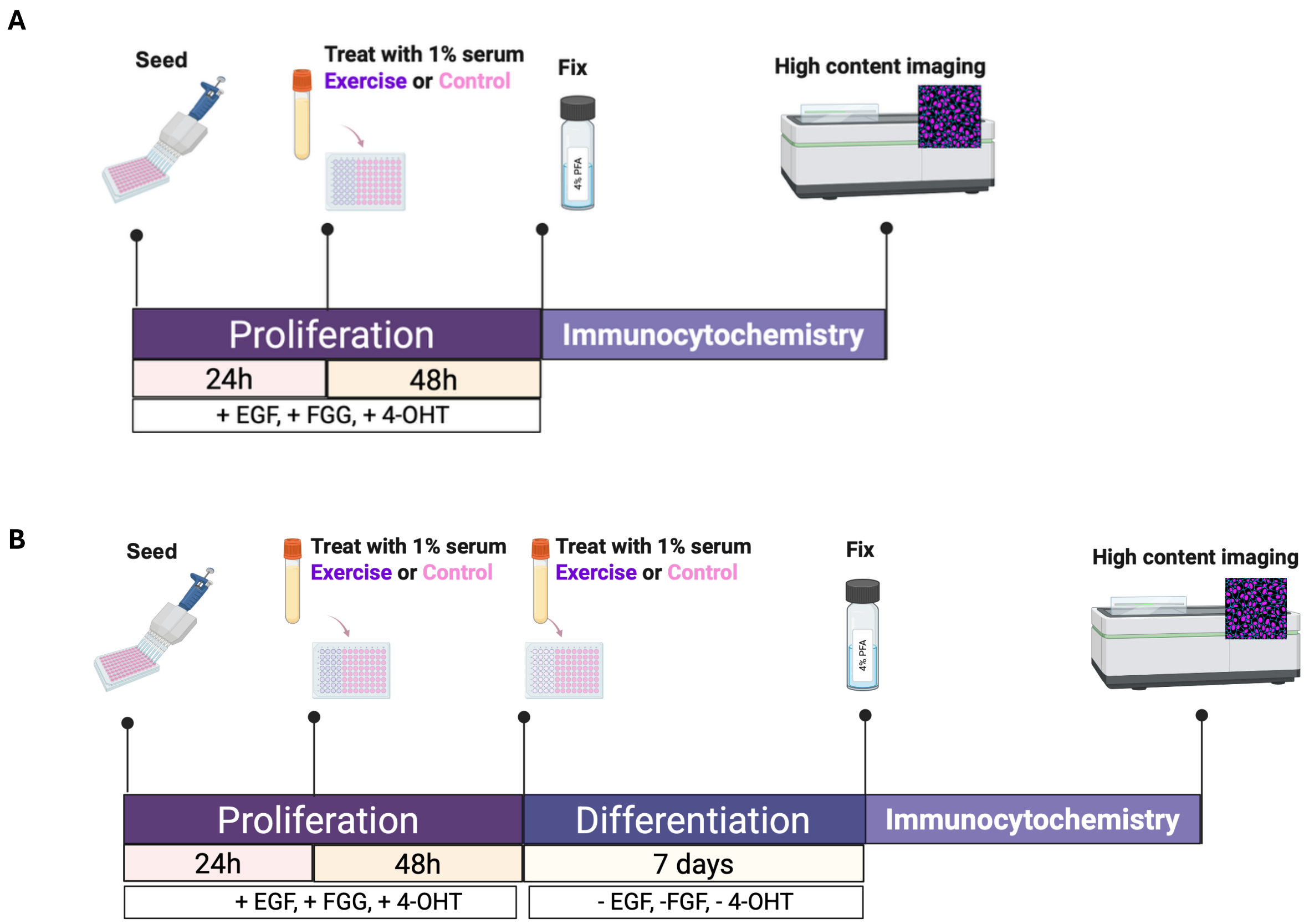

Supplementary Figure 4. *In vitro* parabiosis neurogenesis assay. A) Proliferation phase: Human hippocampal progenitor cells (HPC0A07/03, HPC; ReNeuron Ltd, UK) were plated in proliferation medium containing epidermal growth factor (EGF), fibroblast growth factor (FGF) and 4-Hydroxytamoxifen. 24h after seeding, the medium was replaced with fresh proliferation medium containing 1% serum (collected from exercisers or controls at baseline and endpoint) and 1% penicillin streptomycin. HPCs were subsequently allowed to proliferate for a further 48h, until they were fixed, using 4% paraformaldehyde (PFA), immunostained using the appropriate antibodies (**Supplementary Table 7**) and imaged, using the Opera Phenix high-content imaging system. **B) Differentiation phase:** HPCs were plated under the same initial proliferative condition, in presence of EGF, FGF and 4-OHT. 24h post seeding they were treated with proliferation medium containing; 1% serum and allowed to proliferate for a further 48h. This time, instead of fixation, the proliferation medium was replaced with differentiation media, lacking; EGE, FGF, 4-OHT while containing 1% serum and 1% penicillin streptomycin. HPCs were then allowed to differentiate for 7 days, after which they were fixed in 4% PFA, immunostained (**Supplementary Table 7**), and imaged with the Opera Phenix high-content imaging system. Experiments were performed in three biological replicates, each comprising three technical replicates: passage p21. All quantitative and qualitative image analysis were done on Harmoney software. (Figure created with BioRender).

Supplementary Table 7. List of antibodies used in the *In vitro* parabiosis neurogenesis assay

**Antibody**	**Dilution**	**Manufacturer**	**Cat number**
**Primary**			
Mouse Anti-Ki67	1:800	Cell Signalling	9449
Rabbit Anti-CC3	1:1000	Invitrogen	PA5-114687
Mouse Anti-Nestin	1:1000	Sigma-Aldrich	MAB5326
Rabbit Anti-Sox2	1:1000	Abcam	AB5603
Rabbit Anti-DCX	1:500	Abcam	AB18723
Mouse Anti-Map2	1:500	Abcam	AB11267
**Secondary**			
555 anti-Rabbit	1:500	Invitrogen	A-31572
488 anti-Mouse	1:500	Invitrogen	A-21202
